# The Long-Term Dynamics of Mortality Benefits from Improved Water and Sanitation in Less Developed Countries

**DOI:** 10.1371/journal.pone.0074804

**Published:** 2013-10-08

**Authors:** Marc A. Jeuland, David E. Fuente, Semra Ozdemir, Maura C. Allaire, Dale Whittington

**Affiliations:** 1 Sanford School of Public Policy and Duke Global Health Institute, Duke University, Durham, North Carolina, United States of America; 2 Department of City & Regional Planning, University of North Carolina at Chapel Hill, Chapel Hill, North Carolina, United States of America; 3 Program in Health Services and Systems Research, Duke-National University of Singapore Graduate Medical School, Singapore; 4 Department of Environmental Sciences and Engineering, University of North Carolina at Chapel Hill, Chapel Hill, North Carolina, United States of America; 5 Manchester Business School, Manchester, United Kingdom; University of Florida, United States of America

## Abstract

The problem of inadequate access to water, sanitation and hygiene (WASH) in less-developed nations has received much attention over the last several decades (most recently in the Millennium Development Goals), largely because diseases associated with such conditions contribute substantially to mortality in poor countries. We present country-level projections for WASH coverage and for WASH-related mortality in developing regions over a long time horizon (1975–2050) and provide dynamic estimates of the economic value of potential reductions in this WASH-related mortality, which go beyond the static results found in previous work. Over the historical period leading up to the present, our analysis shows steady and substantial improvements in WASH coverage and declining mortality rates across many developing regions, namely East Asia and the Pacific, Latin America and the Caribbean, Eastern Europe and the Middle East. The economic value of potential health gains from eliminating mortality attributable to poor water and sanitation has decreased substantially, and in the future will therefore be modest in these regions. Where WASH-related deaths remain high (in parts of South Asia and much of Sub-Saharan Africa), if current trends continue, it will be several decades before economic development and investments in improved water and sanitation will result in the capture of these economic benefits. The fact that health losses will likely remain high in these two regions over the medium term suggests that accelerated efforts are needed to improve access to water and sanitation, though the costs and benefits of such efforts in specific locations should be carefully assessed.

## Introduction

The World Health Organization (WHO) estimates that about 780 million people globally live without access to improved water supplies, and 2.5 billion live without adequate sanitation [1], [2]. Diseases associated with poor water, sanitation and hygiene (WASH) conditions comprised 6 to 7% of mortality in less-developed countries in 2008 and remain one of the major contributors to the environmental burden of disease worldwide, despite the recent and significant progress described by the Global Burden of Disease (BOD) project [3], [4], [5]. In fact, WASH-related diseases are largely preventable and are essentially nonexistent in countries with modern piped water and sanitation systems. A growing body of research suggests that various low-cost, non-piped WASH interventions – improved community water supplies, point-of-use water treatment, hygiene education, hand-washing, on-site sanitation – can provide many of the same economic and social benefits, especially health benefits, as piped water and sewer networks [6], [7], [8], [9], and at much lower cost. Even so, many people in less developed countries remain without access to such technologies today, and WASH-related diseases therefore persist.

**Figure 1:**
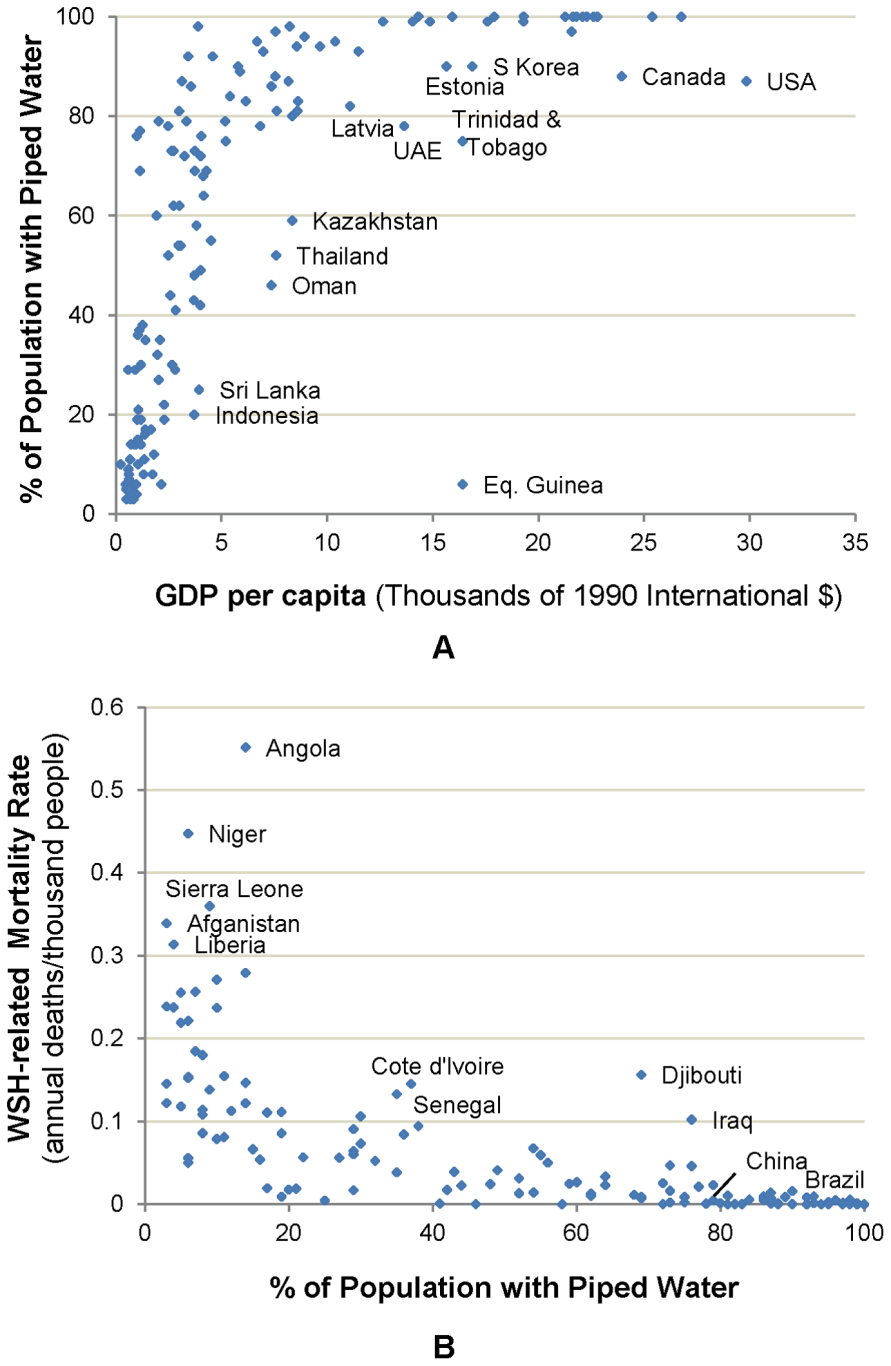
Associations between (A) piped water and average per capita GDP in 2010, and (B) WASH-related death rate and % coverage with piped water in 2004.

The usual depiction of global inadequacy in access to water and sanitation services is static and generally fails to provide perspective on long-term trends in improved WASH coverage. Economic development is clearly positively associated with higher levels of access to improved WASH services and lower WASH-related mortality, as can be seen in both the dramatic increases over time in access to services in fast-growing nations such as China and Brazil, and in cross-country correlations between income and access (Figure 1). Mortality rates from infectious diseases including diarrhea have recently been declining across the developing world, particularly among children, even in places where economic growth has been slow [10]. However, will the current trajectory of economic growth and declining mortality rates solve the health problems associated with inadequate WASH coverage anytime soon? The answer to this question is important because it has implications for how the global community should consider the case for increased future interventions in the water and sanitation sector.

To answer this question, we developed long-range country-level projections (1975–2050) for coverage with three levels of water infrastructure – improved water, improved sanitation (using the WHO definition), and piped water and sewer networks (hereafter called piped services) – as well as mortality rates associated with these coverage levels. We then aggregated these projections into the following six regional groups: East Asia/Pacific (EAP), Middle East (MIDEAST), Latin America and the Caribbean (LAC), Eastern Europe (EURCA), South Asia (SA), Sub-Saharan Africa (SSA). East Asia/Pacific (EAP), Middle East (MIDEAST). The projections were generated using a simulation model that was parameterized using results from cross-country panel regressions of infrastructure coverage and WASH-related mortality. Working with these projections of coverage and health outcomes, we further developed global estimates of the economic value of potential health gains from eliminating mortality from WASH-related diseases. Note that this is not the economic value of the projected year-to-year reductions in WASH-related mortality, but rather the economic value of eliminating the annual remaining, preventable WASH-mortality (hence, we use the term “potential gains” to describe these benefits that could be realized if remaining WASH-related deaths were prevented). We estimate the economic value of eliminating the annual remaining, preventable WASH-mortality changes for each year of the projection period. While complete elimination of such preventable deaths may not be possible, diarrhea mortality data show that WASH-related deaths have been reduced to levels that are practically zero in high-income, industrialized countries. Although these projections are not inclusive of all the economic gains from improving access to water and sanitation services, they do provide important information on the mortality-related consequences of global WASH deficiencies.

This paper does not seek to identify a causal link between average income, coverage with improved water and piped services, and decreases in mortality. Many coincident changes – in infrastructure, technology, quality of health systems, diet and nutrition – occur simultaneously in countries undergoing rapid development. Indeed, as shown in Figure 1, the relationships between coverage with piped services, WASH-related mortality and income are similar to the Preston Curve, which illustrates associations between life expectancy and GDP per capita. This similarity underscores the point that many changes occur during the transition from low to middle-income status [11]. However, the literature offers limited evidence to support a direct, causal interpretation of the relationship between average income, coverage with improved water and piped services, and decreases in mortality, and highlights the problem of reverse causality that may exist in such relationships [12], [13].

Despite the challenges in establishing the causal linkages between these factors, the association is quite robust. For example, using household data, Komives et al. showed that coverage with conventional piped services initially increases very rapidly up to annual household income of about $4,000–$5,000 (1990 International dollars), after which coverage grows more slowly [14]. Günther and Fink find a strong negative association between coverage with improved water and sanitation and mortality rates [15]. Similar relationships between income, coverage, and WASH-related mortality rates hold at the country level (Figure 1). Although these associations do not have a simple causal interpretation, their stability makes them useful for projecting trends in water and sanitation coverage and WASH-related mortality into the future.

By providing a dynamic profile of the economic consequences of WASH-related mortality, the projections in this paper provide a new perspective on the evolving challenge of WASH diseases in poor countries. This work extends beyond the analysis of dynamics in the Joint Monitoring Project (JMP) of the WHO/UNICEF, which do not attempt to project water and sanitation service coverage into the future, or the BOD project of the WHO, which focuses solely on present and future trends in health outcomes, or finally the largely static vision offered by efforts such as the Economics of Sanitation Initiative (ESI) [2], [16], [17]. Our simulations suggest that in many less-developed countries, the changes that accompany economic growth are likely to imply higher levels of coverage with water and sanitation infrastructure and much lower WASH-related mortality rates by 2050, such that economic gains from further mortality reductions will become very modest. On the other hand, for countries in SSA and in parts of SA, low levels of coverage, especially with piped services, seem likely to persist for some time, and WASH-related mortality and its economic consequences will remain substantial even in 2050. The potential economic gains from increased, targeted investments to eliminate WASH-related disease in such countries will likely remain high over the coming decades, but these economic benefits will still need to be carefully compared to the costs of interventions in specific locations [18]. The remainder of the paper describes the methodology used for generating these predictions, presents our results, and discusses their policy implications.

## Methods

**Figure 2:**
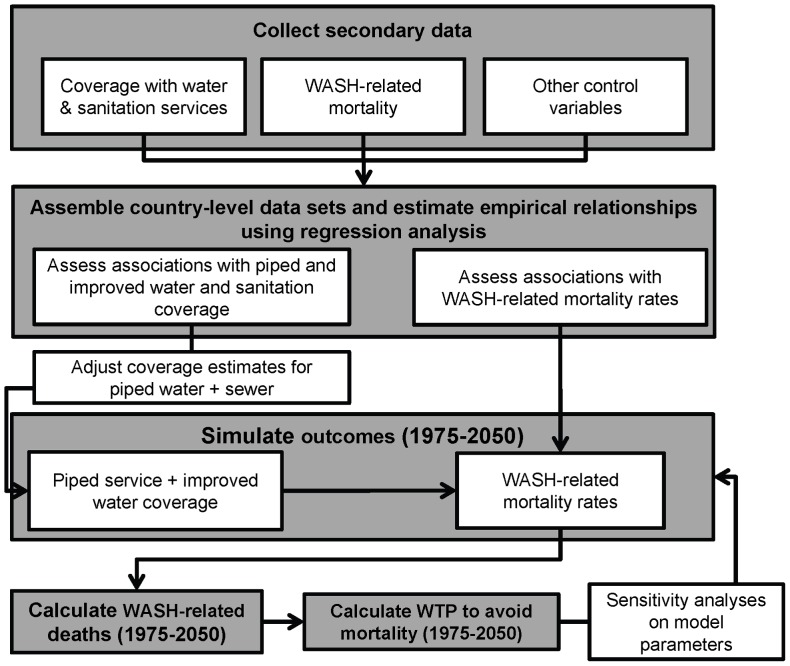
Analytical framework for our calculations of potential economic benefits from eliminating WASH-related mortality.

The analytical framework developed in this study (described in the flow diagram in Figure 2) has three primary components. First, we use econometric techniques to estimate associations between various levels of water and sanitation coverage, income, and urbanization as well as WASH-related mortality rates, income, and water and sanitation coverage. Second, we use these associations along with projections of population growth, urbanization, and economic growth to simulate trends in water and sanitation coverage levels, WASH-related mortality rates, and WASH-related deaths. Finally, we monetize the economic value of potential health gains from eliminating the mortality due to WASH-related diseases using estimates of the value of mortality risk reduction, commonly referred to as the value of statistical life (VSL). We conduct country-level simulations and use country-level data collected from secondary sources whenever possible. We then aggregate results to the regional level for the purposes of presentation, and conduct sensitivity analyses on the core parameters in the simulation model.

As described below, our methodology represents an extension from existing projections of various aspects of the BOD project in two important ways [19], [20]. First, our model focuses only on WASH-related mortality, which allows us to incorporate more complicated dynamics such as changes in income, infrastructure coverage and urbanization than the BOD projections [16]. Second, we estimate the economic value of reducing WASH-related mortality over the projection period.

### Parameterizing the simulation model

To parameterize the simulation model, we use econometric techniques to estimate two equations: 1) the association between various levels of water and sanitation infrastructure coverage (improved water, improved sanitation, and piped water) and income, urbanization, and other country level factors; and 2) the association between WASH-related mortality rates and water and sanitation infrastructure coverage, income, and other country-level factors. Specifically, we estimated the following two equations using panel regression models (OLS, fixed and random effects):
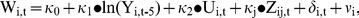
(1)

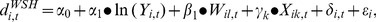
(2)


In equation 1, *W_i,t_* is the water and sanitation coverage in country *i* at time *t*; *Y_i, t-5_* is income (per capita GDP serves as a proxy) from the previous wave of coverage data, 5 years prior (period *t – 5*); *U_i,t_* is the percent of the population that resides in an urban area in country *i* at time *t*; *Z_ij,t_* is a vector of *j* country-level control variables that may be related to coverage (% urban population, income inequality, several governance variables, WHO region and year dummy variables, and a set of time-region interaction variables measured at time *t* for country *i*); *δ_i,t_* is a time-varying error term; and *ν_i_* is a time-invariant error term. We estimate equation 1 for three types of water and sanitation infrastructure coverage: improved water only, improved sanitation only, and piped water. Thus, equation 1 is estimated using regression methods separately for each of these three water and sanitation coverage variables, to yield three sets of *κ* coefficients. We use a five-year lag for income because the infrastructure coverage data were collected at five-year intervals. Sensitivity analyses of model results were conducted for the lag period and for the functional form of the relationship between income and coverage.

In equation 2, 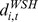 is the WASH-related mortality rate in country *i* and time *t*; *W_il,t_* is now the vector of *l* water and sanitation coverage variables in country *i* at time *t; X_ik,t_* is a vector of *k* country-level control variables that may be related to WASH mortality (% urban population, income inequality, fertility, literacy, child immunization rates, region and year dummy variable, and several governance variables); *δ_i,t_* is a time-varying error term; and *ε_i_* is a time-invariant error term. Thus, equation 2 is estimated as a function of income, the *l* water and sanitation coverage variables, and the *k* other control variables, to yield the *α_n_*, *β_l_*, and *γ_k_* model coefficients. Ideally, equation 2 would control for access to other relevant health services. We use the proxy of child immunization rates with the vaccine against diphtheria, tetanus and pertussis to capture this effect, due to limited data availability (e.g. antenatal care) and concerns about endogeneity between mortality and other potential proxies (e.g. treatment with oral rehydration therapy).

Data used in the regression analysis were assembled from various sources. Table 1 provides summary information for the data included in the simple model with less-developed countries only, as well as the full model including all countries. Mortality attributable to WASH-related diseases was calculated using the methodology developed in the WHO's Environmental Burden of Disease (EBD) project, and more widely applied by the Disease Control Priorities Project (see materials S1 for details on the construction of this variable). Data on coverage with improved water and sanitation services for the period 1990–2010 were obtained from the JMP. Improved water includes plot or yard tap, public tap/standpipe, tube well/borehole, protected dug well, protected spring, rainwater collection, and piped water; improved sanitation includes flush or pour-flush to piped sewer system, septic tank or pit latrine, ventilated improved pit latrine, pit latrine with slab, composting toilet, and sewerage. The Groningen Growth and Development Center (GGDC) database was used for per capita GDP (all normalized to 1990 Geary-Khamis (G-K) dollars); and urbanization rates and population projections came from the UN Population Division [21], [22]. Other country-level variables were also included in testing various model specifications as summarized in Table 1 and described in additional detail in the materials S1 (regional assignments of countries are shown in Table S2). Because of missing data, the regression analyses include between 95 and 150 countries, depending on the model specification and whether high-income countries are included. The majority of missing data were for small island countries.

**Table 1:** Descriptive statistics for variables included in the regression models.

	Simple model (only less-developed countries)					Full model (all countries)					Data source	Data years
Infrastructure coverage models	N	Mean	S. Dev.	Min	Max	N	Mean	S. Dev.	Min	Max		
Piped water coverage (%)	866	53.5	34.2	0	100	970	58.3	35.2	0	100	UNICEF and WHO (2012)	1990,1995,2000,2005,2010
Improved non-piped water cov. (%)	863	27.9	21.8	0	94	967	25.0	22.2	0	94	UNICEF and WHO (2012)	1990,1995,2000,2005,2010
Improved sanitation cov. (%)	881	66.0	31.0	3	100	985	69.6	31.1	3	100	UNICEF and WHO (2012)	1990,1995,2000,2005,2010
5-yr lag GDP per capita (1990 $G-K)	576	3727	3437	218	22515	680	6065	6640	218	36697	Angus Maddison GGDC database; IMF World Economic Outlook	1985–2005
Inequality: % GDP to lowest 80%	615	48.8	8.7	28.5	78.3	707	47.8	8.6	28.5	78.3	World Bank	1990–2010; 1–3x per country
Urbanization (%)	736	50.0	24.3	0	100	840	53.3	24.8	0	100	UNICEF and WHO (2012)	1990,1995,2000,2005,2010
Democracy-Autocracy score^a^	489	1.2	6.8	−10	10	581	2.5	7.0	−10	10	Center for Systemic Peace [32]	1990–2010
Years since last regime change	522	15.5	17.6	0	93	614	23	30	0	196	Center for Systemic Peace	1990–2010
Coup (coup in last 5 years)	946	0.18	0.38	0	1	1050	0.16	0.37	0	1	Center for Systemic Peace	1990–2010
WASH Mortality models	N	Mean	S. Dev.	Min	Max	N	Mean	S. Dev.	Min	Max		
WASH-related death rate (per 1,000) b	423	0.46	0.64	0	4.33	501	0.39	0.61	0	4.3	Estimated from WHO 2004, 2010	2002, 2004, 2008
Piped water coverage (%)	504	54.1	33.5	2	100	582	60.1	34.8	1	100	UNICEF and WHO (2012)	Multiple years c
Improved non-piped water cov. (%)	504	28.8	21.2	0	75	582	25.1	22.7	0	94	UNICEF and WHO (2012)	Multiple years^c^
Improved sanitation coverage (%)	513	67.1	30.8	7	100	591	71.4	30.2	7	100	UNICEF and WHO (2012)	Multiple years^c^
5-yr lag GDP per capita (1990 $G-K)	426	3674	3295	213	16177	504	6149	6767	213	34201	Angus Maddison GGDC database; or IMF [33] when missing	1997–2003
Inequality: % GDP to lowest 80%	357	49.1	8.3	28.9	78.3	426	47.8	8.4	28.9	78.3	World Bank	1990–2010; 1–3x per country
Urbanization (%)	470	51.1	22.7	0	94	548	54.9	23.4	0	100	UNICEF and WHO (2012)	2002–2008
Literacy (% of adults)	368	79.7	20.1	14.2	99.9	446	83	20	14	100	UNESCO	2002–2008
Child DTP-3 coverage (%)	499	83.9	17.1	19	99	577	85	16	19	99	WHO	2002–2008
Democracy-Autocracy score^a^	388	2.4	6.6	−10	10	457	3.5	6.6	−10	10	Center for Systemic Peace	2002–2008
Years since last regime change	411	17.4	18.4	0	97	480	25.3	30.9	0	199	Center for Systemic Peace	2002–2008

a Extent of democracy and autocracy in a country. Score of +10 indicates strongly democratic, while −10 is strongly autocratic.

b Estimated using methodology of WHO Environmental Burden of Disease.

c Interpolated between years of available data.

### The simulation model and sensitivity analysis

The simulation model combines the results of the regression analyses described above with projections of economic growth, urbanization, and population. The simulation model projects the following four variables over the simulation horizon (1975–2050) for 140 less-developed countries for which we have income, coverage and WASH-mortality data: 1) water and sanitation coverage levels, 2) WASH-related mortality rates, 3) WASH-related deaths, and 4) potential economic gains from eliminating WASH-related mortality. Only one outlier country for which we have data – Equatorial Guinea – is excluded, because this oil-rich country does not appear to follow the trajectory observed in other countries, as income is high, water and sanitation coverage rates are low, and mortality is very high. The equations used to project these trends and input data are described in more detail below.

The model combines the empirical estimates of the association between water and sanitation coverage from the regression analysis with projections of GDP and urbanization to forecast future water and sanitation coverage (equation 3):
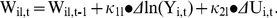
(3)


In equation 3, *W_il,t_* is the percent coverage of water and/or sanitation service level *l* (improved water, improved sanitation, or piped water) in country *i* at time *t*; *Δln (Y_it_)* is the difference between the natural log of per capita GDP in time *t* and the natural log of per capita GDP at time *t-1* in country i; *ΔU_it_* is the projected change in urbanization from time *t* to *t-1*; *κ_1l_* is the parameter estimate from equation 1 that describes the association between income and coverage with water and sanitation service level *l*; and *κ_2l_* is the parameter estimate from equation 1 that describes the association between urbanization and coverage with water and sanitation service level *l*. In our base case analysis, the input data for changes in GDP are based on historical country-level income trends for 1950–2010. For simulated estimates of piped water plus sewerage, we adjust the piped water estimates downwards by 12% for all countries in which piped water coverage is greater than 20%, and by a linear factor down to no difference at a coverage level of zero for those in which piped water coverage is less than 20%, based on average differences between piped water and piped water and sewerage estimates presented in Komives et al. [14].

The model then combines our estimates of the association between WASH-mortality rates from our regression analysis with projections of access to water and sanitation services (obtained using equation 3), income and population to estimate the annual number of deaths attributable to WASH-related diseases in each of the 140 countries included in the simulation (equation 4).
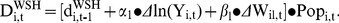
(4)


In equation 4, 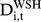 is the number of deaths attributable to the lack of access to water and sanitation services in country *i* in year *t* (and 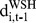 is the death rate in year *t-1*); *Δln (Y_i,t_)* is the difference between the natural log of per capita GDP in time *t* and the natural log of per capita GDP at time *t-1* in country *i*; *ΔW_il,t_* is the change in water and sanitation coverage level *l* between times *t* and *t-1* in country *i*; 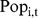 is the population in country *i* at time *t*; *α*_1_ is the parameter estimate from equation 2 that describes the association between the WASH mortality rate and income; and *β_l_* is the parameter estimate from equation 2 that describes the association between water and sanitation service *l* (improved water, improved sanitation, or piped water) and the WASH mortality rate.

To value the potential economic benefits from eliminating mortality attributable to poor water and sanitation, we utilized the concept of the economic value of mortality risk reduction based on individuals' willingness to pay (WTP), commonly referred to as the value of a statistical life (VSL), to monetize reductions in WASH-related mortality risk [23], [24]. The VSL is a willingness-to-pay measure derived by economists based on the pecuniary tradeoffs individuals are willing to make between income and small changes in mortality risk. We model the relationship between the VSL and income using a function developed on the basis of the best estimates of the VSL available in the literature from less-developed countries. Description of the specific method used to estimate the VSL in this study is provided in the accompanying materials S1 (see also Tables S1 and Figures S1–S2).

Our model estimates the potential economic benefits of eliminating WASH-related mortality by multiplying the VSL by the projected number of WASH-related deaths in each country in year t (equation 5).
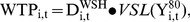
(5)


In equation 5, *WTPi,t* is the potential economic benefit from elimination of mortality due to WASH-related diseases in country *i* in year *t*; 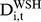 is the total number of WASH-related deaths in country *i* in year *t* from equation 4; and *VSL
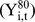
* is the value of statistical life in country *i* in year *t*. Because low-income individuals are most likely to suffer from WASH-related mortality, we use the per capita GDP of the bottom 80^th^ percentile for each country to obtain more appropriate and conservative estimates of the VSL for each country.

To address inconsistencies that likely result from measurement error in the original data, our graphical results show 5-year moving averages for all projections. For the analysis, we subjected our projections to a number of sensitivity analyses to assess the extent to which alternative assumptions affect our results. Assumptions examined in our sensitivity analysis included but were not limited to the following: 1) the magnitude of the associations between income, urbanization, coverage rates, and WASH-related mortality; 2) changes in the assumed GDP growth trajectory (using short term (1990–2010) versus long-term (1950–2010) GDP growth); and 3) the nature of the relationship between the VSL and income. The scope and results of these sensitivity analyses are summarized in detail in the accompanying materials S1.

## Results

### Calibration of model relationships

Regression results for equation 1 with piped water as the outcome variable are reported in Table 2 (as documented in Tables S3, S4, S5, results for coverage with improved water and improved sanitation are similar, though urbanization is less significant for improved water). These models are estimated using data from 95 less-developed and 36 high-income countries. A 1-log increase in per capita 5-year lagged GDP is associated with an 8 (fixed effects model) to 11% (random effects model) increase in piped water coverage in the current period. Percent urban population is also significant and positive – a 0.37–0.45% increase in piped coverage for a 1% percentage increase in urban population. We use the 95% confidence intervals from these estimated associations in our simulation model. Regional indicator variables (for the six less-developed regions) for all regions except EAP are significant and positive relative to SSA, and time period indicators (for the year to which the data pertain) suggest there may have been accelerating expansion of services over time. Finally, the democracy-autocracy variable is marginally significant and negative in both specifications, suggesting that more democracy is associated with lower coverage, but none of the other governance or inequality variables have significant associations with piped water coverage.

**Table 2:** Estimations of country-level coverage with piped water.

	Random Effects a		Fixed effects	
	Simple model	Full model (all countries) b	Simple model	Full model (all countries) b
5-yr lagged ln GDP per capita	11.2*** (1.96)	9.9*** (1.83)	8.1*** (2.2)	6.1*** (2.1)
% of GDP to lowest 80% of population	0.11 (0.10)	0.11 (0.09)	0.12 (0.11)	0.13 (0.10)
% Urban population	0.45*** (0.08)	0.48*** (0.08)	0.37** (0.17)	0.45*** (0.16)
Countries in LAC region	27.9*** (5.0)	27.4*** (4.9)		
Countries in MIDEAST region	30.8*** (5.6)	31.6*** (5.4)		
Countries in SOUTH ASIA region	5.0* (2.7)	5.6** (2.8)		
Countries in EAST ASIA/PACIFIC region	8.2 (5.2)	8.9* (5.3)		
Countries in EASTERN EUROPE region	44.4*** (4.2)	32.9*** (4.3)		
Developed countries		37.5*** (6.2)		
1990	−4.7*** (1.4)	−2.9** (1.2)	−6.4*** (1.8)	−4.7*** (1.7)
1995	−2.6** (1.1)	−0.64 (0.81)	−4.0*** (1.4)	−2.0* (1.2)
2000	−0.85 (0.78)	−0.78 (0.69)	−1.9** (0.97)	−1.7* (0.91)
2005	0.21 (0.38)	−2.3*** (0.61)	−0.38 (0.49)	−2.6*** (0.65)
Democracy-Autocracy Score	−0.16* (0.10)	−0.056 (0.09)	−0.23** (0.10)	−0.20* (0.10)
Years since last regime change	−0.013 (0.05)	−0.050 (0.04)	−0.027 (0.05)	−0.066** (0.05)
Coup	−1.3 (1.1)	−1.6 (1.1)	−0.93 (1.2)	−1.2 (1.1)
Constant	−80.1*** (12.1)	−72.3*** (11.7)	−37.0** (17.7)	−19.9 (19.3)
Number of observations	470	634	470	634
Number of countries	95	131	95	131
Adjusted R^2^ (overall)(within)(between)	0.9010.5510.913	0.9070.4890.914	0.7740.5590.800	0.7910.5020.808
Hausman Test for simple model *χ* ^2^ (p-value)	78.2 (0.000)			

Notes: *Significant at 90%, **Significant at 95%, ***Significant at 99%. Robust standard errors. The omitted region is SSA; the omitted year is 2010.

a A random-effects tobit model with censoring at 0 and 100% coverage does not yield qualitatively different results.

b Includes all countries (including developed and former Soviet republics dropped from the simple model) and full set of year-region interactions.

The results of the regressions for WASH-related death rates (equation 2) are presented in Table 3. These models are estimated using data from 129 less-developed and 23 developed countries. Column 1 presents results from a simple model specification that includes all countries and relates mortality rates directly to income without including coverage variables (this specification is not used for our projections because we are interested in the relationships between service coverage, mortality rates, and their economic consequences). Columns 2–5 correspond to the structural form of equation 2 and provide evidence that piped water and improved water coverage has significant and negative associations with the WASH death rate. The magnitude of these associations may be slightly greater for non-piped improved water, and are similar whether or not developed countries are included in the model. A 1% increase in piped water or improved water coverage is associated with a decrease of 0.015–0.025 deaths per thousand people per year due to WASH-related diseases (we use 0.02 in the base case for our simulations). Somewhat surprisingly, coverage with improved sanitation does not appear to be significantly associated with the WASH-death rate. This may be due to high correlation (over 0.8) with the water coverage variables.

**Table 3:** Estimation of WASH-related mortality (deaths per thousand people per year)^a^.

	All countries, reduced form (random effects) a		All Countries, base model (random effects) a		All Countries, base model (fixed effects) b		Less-developed countries only, base model random effects)		All Countries, full model (random effects)	
	Coef.	Std. Err.	Coef.	Std. Err.	Coef.	Std. Err.	Coef.	Std. Err.	Coef.	Std. Err.
% Piped water coverage			−0.024***	0.0051	−0.023**	0.011	−0.024***	−0.0052	−0.015***	0.0051
% Improved non-piped water coverage			−0.028***	0.0056	−0.035***	0.011	−0.027***	−0.0056	−0.017***	0.0046
% Improved sanitation coverage			−0.0001	0.0032	−0.012	0.011	−0.00013	0.0033	0.0061*	0.0032
5-yr lagged ln per capita GDP	−0.30***	0.07	−0.20***	0.076	0.038	0.18	−0.20***	−0.079	−0.14	0.094
% Urban population	−0.0027	0.0025	0.0029	0.0030	−0.026	0.022	0.0033	0.0033	0.00082	0.0030
Literacy									−0.014***	0.0036
% of GDP to lowest 80% of population									0.0024	0.0054
Child vaccination – Dtp3									−0.012***	0.0025
Developed countries	−0.52***	0.19	−0.41***	0.16					−0.23	0.27
Countries in LAC region	−0.82***	0.14	−0.67***	0.13			−0.68***	0.13	−0.54***	0.18
Countries in MIDEAST region	−0.81***	0.17	−0.61***	0.17			−0.62***	0.17	−0.66***	0.24
Countries in SOUTH ASIA region	−0.79***	0.16	−0.20	0.16			−0.21	0.16	−0.52**	0.23
Countries in EAST ASIA/PACIFIC region	−0.92***	0.14	−0.72***	0.14			−0.73***	0.14	−0.58***	0.18
Countries in EASTERN EUROPE region	−0.90***	0.14	−0.58***	0.14			−0.59***	0.15	−0.37*	0.21
Democracy-Autocracy Score	−0.0040	0.0059	0.0006	0.0059	−0.0071	0.0073	0.0016	0.0058	0.0034	0.0068
Years since last regime change	0.0013	0.0015	0.0012	0.0014	0.0031*	0.0018	0.0017	0.0023	−0.0001	0.0016
2004	0.021	0.027	0.038	0.028	0.058*	0.030	0.042	0.034	0.084***	0.031
2008	−0.11***	0.031	−0.070**	0.027	−0.012	0.047	−0.090***	0.033	−0.0067	0.035
Constant	3.7***	0.50	4.4***	0.65	−4.6**	2.3	4.4***	0.67	4.9***	0.67
Number of observations	460		451		451		382		354	
Number of countries	155		152		152		129		127	
R^2^ (within)	0.096		0.164		0.209		0.172		0.212	
R^2^ (between)	0.675		0.741				0.722		0.780	
R^2^ (overall)	0.633		0.699				0.679		0.738	

Notes: *Significant at 90%, **Significant at 95%, ***Significant at 99%. Robust standard errors. The omitted region in these regressions is Sub-Saharan Africa (SSA); the omitted year is 2002.

a A Breusch-Pagan specification test is highly significant (P-value<0.000), indicating that the random effects model is more efficient than a standard OLS specification.

b Hausman tests indicate that we cannot reject the hypothesis that the model estimates obtained from random effects and OLS (P-value  = 0.99), and fixed effects and OLS (P-value  = 0.38) specifications, are systematically different. The Hausman test however suggests that random effects and fixed effects estimates are different in the basic model (P-value  = 0.0066) but not in the full model; also, see caveats in text.

A 1-log increase in 5-year lagged income is associated with a decline in WASH-related deaths of 0.15–0.3 per thousand per year; this effect of income excludes its indirect association with coverage with improved services. The negative association between mortality and income holds except in the fixed effects model, and in the random effects model that includes additional controls (perhaps because of the high correlation between income and literacy, which becomes significant). There are reasons to suspect that the fixed effects results may be unreliable, due to inconsistencies in measurement as well as lack of variation within countries in the short time series for mortality. For example, the WHO cautions that “estimates for different years are not directly comparable because they include: (1) varying vital registration data, (2) different sources of epidemiological data for specific causes, and (3) information on child and adult mortality that varies by year in countries lacking good death registration data. In addition, there may be errors in the data. For example, the WHO data for India indicate many more deaths from diarrhea in 2008 relative to 2002 and 2004, but closer inspection reveals that this increase is for adults, which seems implausible. Measurement error has much greater influence on the fixed effects estimations that rely on within-country variation for identification. Since income was generally increasing over time in less-developed countries throughout the period 2002–2008, systematic measurement errors of this type are largely absorbed by the income term and the time period indicators.

Finally, percent urban population, inequality, the democracy-autocracy index, and the number of years since a regime change do not have consistently significant associations with the WASH death rate across specifications. The associations with literacy and with child vaccination rates, however, are negative and significant; higher education and vaccination levels thus appear correlated with lower death rates. Inclusion of vaccination rates slightly decreases the associations between water service coverage and WASH-related mortality.

We also estimated additional models to test the sensitivity of these results to assumptions about the functional form (linear and squared terms) and lag structure of the relationships between coverage, mortality, and income variables. These additional analyses, detailed in Tables S3 and S6, provide some evidence that expansion of piped services slows when GDP is high, once coverage reaches high levels. These alternative specifications suggest that there may be: 1) a slowing of expansion of piped and improved services with GDP after an initial rapid increase; 2) a concave relationship between death rates and piped water coverage, whereby declines in WASH-related deaths decrease as coverage increases; 3) a linear relationship between declines in WASH-related deaths and coverage with other improved water sources; and 4) limited association between country-level improved sanitation coverage and the WASH-related death rate independent from that with water services coverage. The associations between coverage, mortality and lagged income are significant and consistent across specifications. Similar tests on the inclusion of different income terms also suggest that the log income specification outperforms models with linear or higher order income terms, and that model fit declines moderately when shorter lags (1 or 3 years) are used.

### Progression of water and sanitation coverage across developing regions

**Figure 3:**
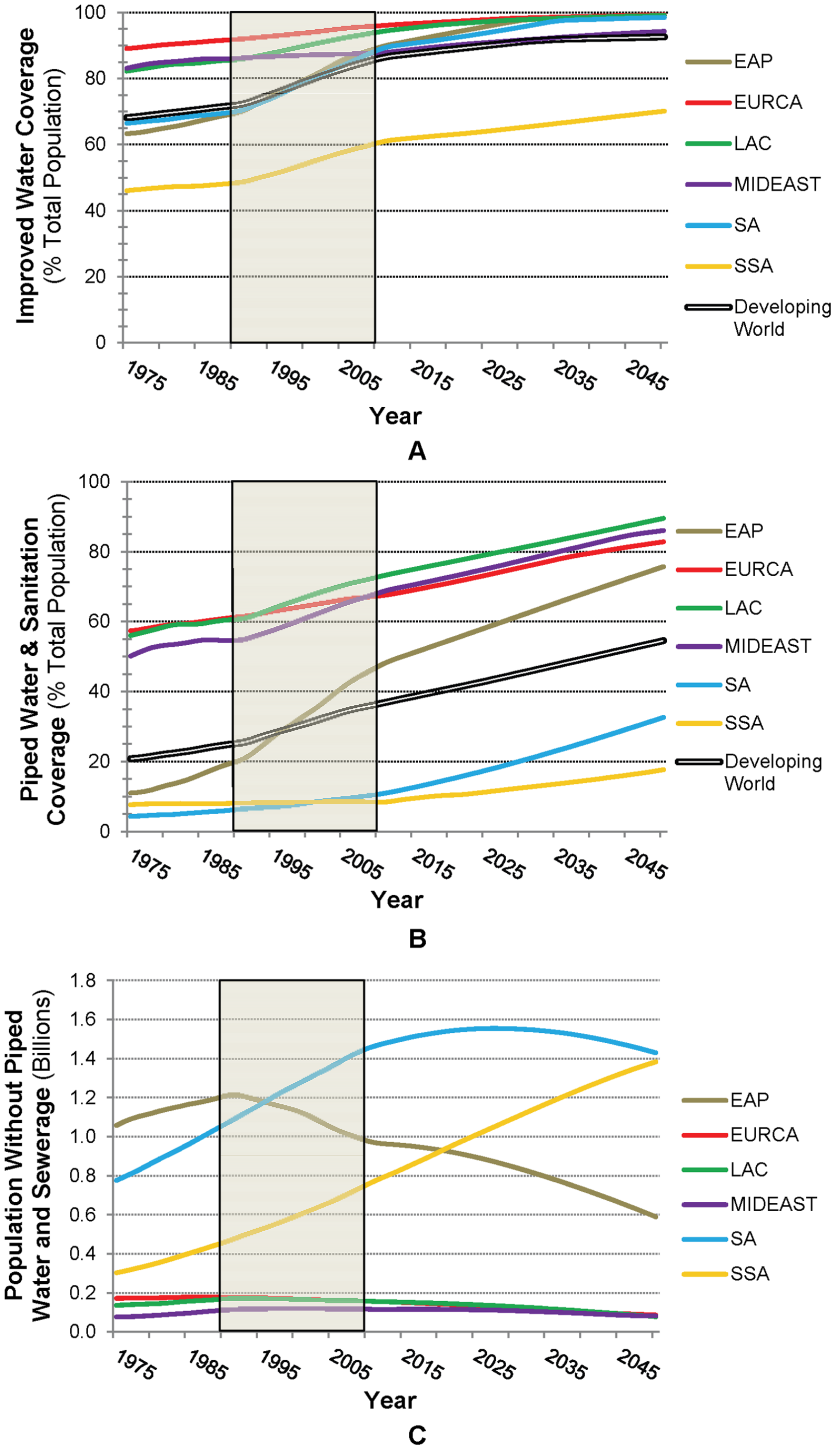
Coverage with improved water, shown for (A) % of overall population; (B) % with piped water and sewerage only; and (C) total population without piped water and sewerage, by region. (Data from WHO/UNICEF JMP project; actual data period is shown by shaded box; future projections use long term historical growth rates.) The bold black and white line in Panels A and B represents the population-weighted average across less-developed countries.

On the basis of the regression results discussed in the previous section, we specified the simulation model parameters as summarized in Table 4, and then used these to simulate outcomes according to equations 3–5. With regards to coverage with water and sanitation services (equation 3), we find a gradual increasing trend in access to improved water and piped services (percent of population covered) across developing regions. For improved water, coverage in developing regions rises from about 68% in 1975 to 93% in 2050 (Figure 3, Panel A); for piped services the change is from 21% to 55% (Panel B). In 2050, coverage levels are lowest in SSA – 70% for improved water and 18% for piped services. Coverage with piped services is also very low in SA throughout the simulation period. The most dramatic expansion of coverage with both improved and piped services occurs in EAP, especially between 1990 and 2010. This rapid expansion is mostly due to recent progress in China.

**Table 4:** Summary of key simulation model parameters and assumptions.

Parameter	Base case	Range
Equation 3: Service coverage		
-Log-income elasticity κ_1_ (piped water)	9.5	4.5–14.5
-Log-income elasticity κ_1_ (improved water)	4.5	0.0–9.0
-Log-income elasticity κ_1_ (improved sanitation)	8.0	2.0–14.0
-Urbanization elasticity κ_2_ (piped water)	0.35	0.10–0.60
-Urbanization elasticity κ_2_ (improved water)	0.30	0.10–0.50
-Urbanization elasticity κ_2_ (improved sanitation)	0.45	0.10–0.80
-Income projections	Long-term growth^c^	Long or short-term growth^c^
Equation 4: WASH-related mortality		
-Log-income elasticity α_1_	−0.2	−0.3-(−0.1)
-Inclusion of improved water	Yes	Yes or No
-Coverage elasticity β_1_ (piped water)	−0.02	−0.03-(−0.01)
-Coverage elasticity β_1_ (improved water)	−0.0275	−0.045–0
-Coverage elasticity β_1_ (improved sanitation)	0	Not included
-Income projections	Long-term growth (1950–2008)	Long or short term (1990–2008)
Equation 5: Economic benefits		
-VSL specification	Empirical model b	Empirical model b or Income elasticity range 1.5–2
-Income projections a	Long-term growth (1950–2008)	Long or short term (1990–2008)

a In the base case, we use average income among the bottom 80% to calculate the economic benefits from mortality reductions; in sensitivity analysis we also explore using overall average income (see Figure S3 for one-way effects of different parameter assumptions on results).

b Details of the empirical model for the VSL are available in the materials S1.

c Long-term growth corresponds to growth over the period 1950–2008; short-term is over the period 1990–2008.

Despite the upward trend in percent coverage, the absolute numbers of people globally without piped water and sewerage is projected to continue increasing until about 2025 due to population growth. This trend is driven by coverage not keeping pace with population growth in SSA and in SA (Figure 3, Panel C), though population without coverage in other regions is now declining. The base case projections suggest that 1.2 billion people in SSA and 1.4 billion in SA could remain without piped services in 2050. The total number of people without piped services for these two regions in 2050 is forecast to be higher than it is currently, such that the roughly 3.7 billion people lacking piped services globally today is projected to remain unchanged in 2050. In contrast, the total population not covered with improved water declines over the entire simulation period in all regions except SSA, where it continues to increase through 2050.

Our projections of coverage rates and population without access to services are somewhat sensitive to assumptions about the strength of association between coverage with the different categories of water and sanitation services and GDP growth (see Table 5, which also includes improved sanitation). The base case analysis suggests that the percent coverage will increase by 7%, 18%, and 18% for improved water, improved sanitation, and piped services, respectively, between 2010 and 2050. However, sensitivity analysis shows that coverage increases could be as low as 1–7% across these service levels (if economic growth is slow and the associations between growth and coverage are weak), or as high as 9–30% (if growth is high and these associations are strong).

**Table 5:** Summary of projected coverage with different levels of water and sanitation services.

	Improved water only	Improved sanitation only	Piped water	Piped water + sewerage
**Worldwide coverage** (%)				
-Year 1975 Estimate	68 (65–71)	34 (31–38)	30 (27–34)	21 (18–24)
-Year 1990 Data	72	40	36	25
-Year 2010 Data	86	57	47	37
-Projection 2050	93 (87–95)	75 (63–83)	65 (54–74)	55 (43–67)
**Population unserved** (billions)				
-Year 1975 Estimate	1.0 (0.9–1.1)	2.1 (2.0–2.2)	2.3 (2.1–2.4)	2.6 (2.5–2.6)
-Year 1990 Data	1.2	2.6	2.8	3.3
-Year 2010 Data	0.8	2.5	3.1	3.7
-Projection 2050	0.6 (0.4–1.1)	2.0 (1.4–3.0)	2.9 (2.1–3.8)	3.7 (2.7–4.7)

Notes: Low and high estimates in parentheses are derived using the ranges of parameters shown in Table 4.

### Trends in WASH-related mortality

**Figure 4:**
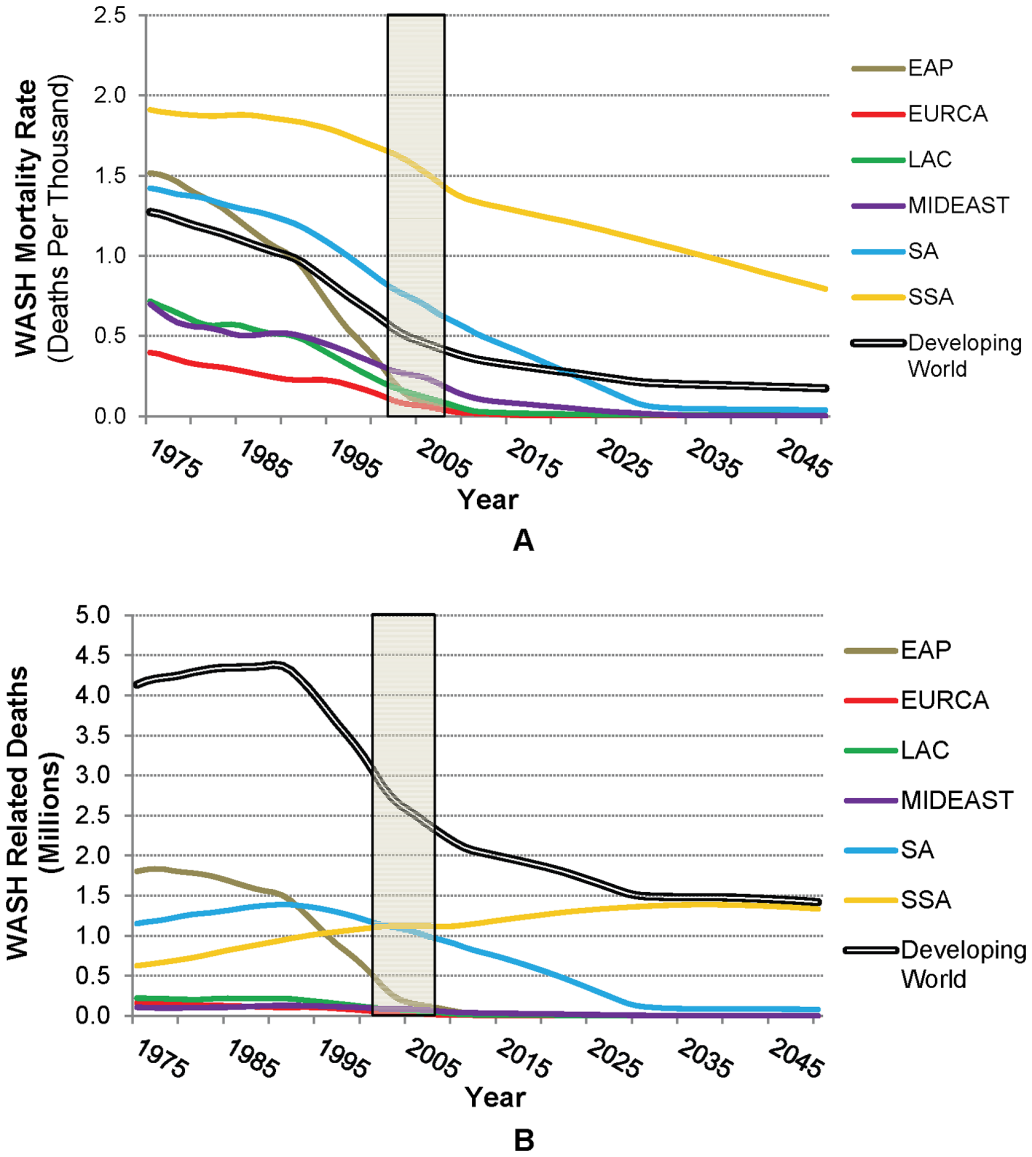
WASH-related disease burden in terms of (A) population-weighted mortality rate and (B) estimated number of deaths, by region (Data for 2002–2008, obtained from the WHO, are shown by the shaded area; future projections use historical GDP growth rates). The bold black and white line in Panel A represents the population-weighted average across less-developed countries, whereas that in Panel B is the total number of deaths in less-developed countries.

The simulation model also projects decreasing WASH-related mortality rates over much of the historical period and in the future (Figure 4). In fact, as shown by the historical data, WASH-related mortality rates in many developing regions have already fallen to low levels except in SA and SSA. The average WASH-related mortality rate was 0.41 deaths per thousand in less developed countries in 2008, down from an estimated 1.3 in 1975, with a projected further decrease to 0.2 by 2050. Throughout the simulation period, mortality rates remain highest in SSA, where economic growth and service expansion are forecast to grow most slowly. The simulated decrease to very low WASH-related mortality rates occurs earliest in EURCA, followed by EAP, LAC and MIDEAST. The decline is steepest in EAP (driven by falling mortality rates in China). WASH-related mortality is projected to remain substantial in SA until about 2030.

The annual number of WASH-related deaths follows an inverted U-shaped curve in several regions, because population growth must slow sufficiently before the general decline in the mortality rate described above reduces the number of total deaths. The peak in these deaths is simulated to have occurred in 1976 in EAP and 1990 in SA, while deaths are estimated to have been declining in EURCA, LAC and MIDEAST throughout the 1975–2050 period (Figure 3). WASH-related deaths in SSA, however, are projected to increase from 1.1 million (today) to a peak of 1.4 million in 2037, at which time 94% of deaths from WASH-related illnesses globally are projected to be in SSA. In 2050 the only other region with significant predicted numbers of WASH-related deaths is SA, and these deaths are mostly in Afghanistan, where mortality rates (2.4 deaths per thousand in 2008) are currently at levels comparable to those in the SSA countries with the highest mortality (Angola, Chad, and Niger).

The simulated future mortality rates and estimated WASH deaths are very sensitive to model assumptions (Table 6). In 2050 average global WASH-related mortality could range from 0.08 to 0.36 deaths per thousand. The implied number of deaths from WASH-related disease could thus range from 0.6 million per year (best case) to 3.0 million per year (worst case) in 2050 (see the materials S1 for additional discussion of the main drivers of this variation in projected WASH-related mortality rates).

**Table 6:** WASH-related mortality and its economic consequences.

	Year				
	1975 Estimates (Simulated)	2002 Estimates (from WHO data)	2008 Estimates (from WHO data)	2050 Projections (Simulated)	Total for future (2012–2050)
**WASH-related Mortality rate** (deaths/thousand)					
All developing regions	1.3 (0.6–1.3)	0.55	0.41	0.2 (0.08–0.4)	n.a
South Asia (SA)	1.4 (0.9–2.5)	0.79	0.61	0.04 (0.0–0.4)	n.a
Sub-Saharan Africa (SSA)	1.9 (1.6–2.4)	1.6	1.4	0.8 (0.6–1.3)	n.a
**WASH-related deaths** (millions)					
All developing regions	4.1 (2.0–7.2)	2.9	2.3	1.4 (0.6–3.0)	64 (29–104)
South Asia (SA)	1.2 (0.7–2.0)	1.1	0.97	0.3 (0.0–0.7)	11 (1.9–35)
Sub-Saharan Africa (SSA)	0.6 (0.5–0.8)	1.1	1.1	1.3 (0.6–2.2)	52 (26–66)
**Economic benefits of eliminating WASH-related mortality** (billions of $US)	(Simulated)	(Simulated)	(Simulated)	(Simulated)	(Simulated)
All developing regions	71 (34–142)	52 (47–176)	42 (41–194)	26 (0.0–1,149)	698 (283–10,306)
East Asia & Pacific (EAP)	30 (9.6–83)	7.8 (5.6–45)	2.4 (2.3–28)	0.0 (0.0–5.6)	2.4 (0.4–234)
Europe & Central Asia (EURCA)	3.5 (1.0–32)	1.0 (0.4–7.9)	0.4 (0.4–5.2)	0.0 (0.0–0.4)	0.8 (0.4–27)
Latin America & Caribbean (LAC)	5.3 (1.9–44)	2.5 (1.2–20)	1.2 (1.1–13)	0.1 (0.01–6.9)	3.3 (1.1–101)
Middle East & North Africa (MENA)	2.1 (1.2–21)	1.8 (1.3–12)	1.3 (1.2–10)	0.0 (0.0–24)	7.0 (3.9–249)
South Asia (SA)	19 (11–79)	20 (18–62)	18 (17–97)	1.5 (4.4–862)	165 (42–7,304)
Sub Saharan Africa (SSA)	11 (8.9–80)	18 (18–28)	19 (19–41)	24 (4.4–251)	519 (235–2,391)

Notes: Aggregated future gains are discounted at 3%, year-specific estimates for 2050 are undiscounted.

Base case followed by low and high projections in parentheses. Low, base and high case estimates are derived using the ranges of model parameters shown in Table 4. Base case assumes linear associations between coverage and mortality rates and includes the effect of improved water coverage as well as piped water coverage.

### Economic gains from eliminating WASH-related mortality

**Figure 5:**
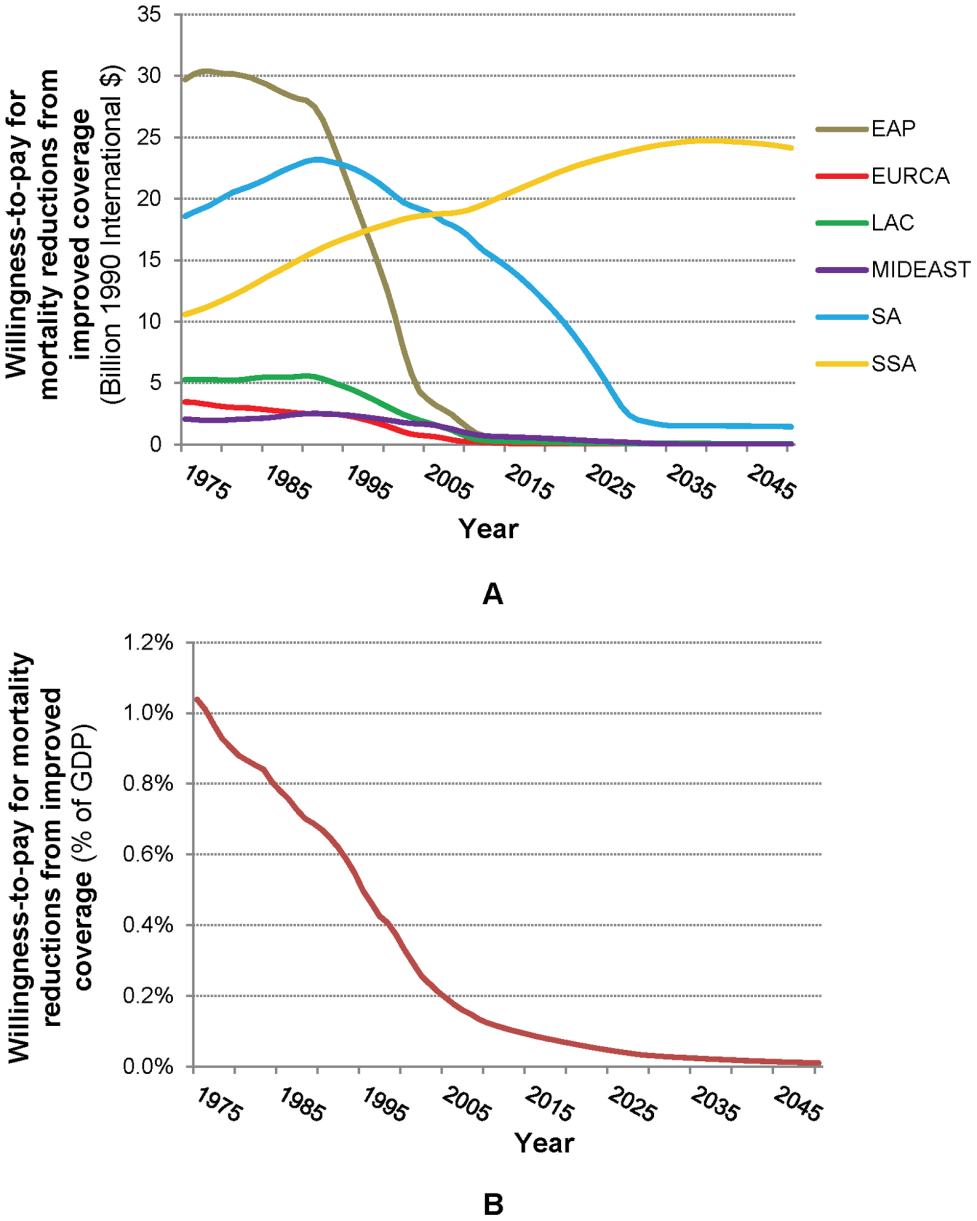
Potential gains from avoiding WASH-related mortality (A) in 1990 International G-K Dollars, and (B) as % of global GDP (projections from historical economic growth rates).

The regional trends in potential economic benefits from eliminating mortality attributable to poor water and sanitation follow an even more pronounced inverted U-shaped trajectory than do those in WASH-related deaths (Figure 5, Panel A). Only in EURCA do these potential economic benefits consistently decline over the simulation period. We simulate a very pronounced peak in EAP ($30.4 billion, in 1978), followed by LAC ($5.6 billion, in 1990), MIDEAST ($2.5 billion, in 1991) and SA ($23.2 billion, in 1992). SSA only reaches its peak of $24.7 billion in 2040; these then decline only slightly to $24.1 billion by 2050. Globally, in the base case projections, we estimate that the potential annual gains began at $71 billion in 1975, peaked at $78 billion in 1990, and will decline to $26 billion by 2050 (from about $37 billion today).

To understand the inverted U-shaped pattern in the simulation results for potential annual economic benefits from eliminating WASH-related mortality, one must consider the various factors that enter into these calculations. Several aspects of the trajectory contribute to increases in these potential benefits (i.e., the rising side of the trajectory). First, there is a positive relationship between income and willingness-to-pay to avoid mortality. When this positive relationship is combined with the positive trend in real income in developing regions between 1975 and 2050, the result is a rising economic value of avoiding WASH-related mortality *per death* (i.e. the VSL). This rising economic value of WASH-related mortality risk reductions has largely been overlooked in the literature on water and sanitation. Somewhat counter-intuitively, it implies that the economic benefits of eliminating WASH-related mortality may not consistently decrease even when the number of deaths is falling, as the “cost” of each death increases in relative terms. The EURCA region presents an interesting exception to the consistent upward trend in benefits per death avoided because real incomes declined in many of these countries in the early 1990s after the fall of the Soviet Union. Similarly, countries that have experienced short-term declines in real income (perhaps due to internal strife or other economic crises) also exhibit declines in the value of mortality risk reductions. Population growth is another contributing factor, because the number of deaths sometimes increases even while mortality rates decline (Figure 4). Indeed, given the positive trend in regional real income, aggregate (as opposed to per capita) economic gains from eliminating these mortality risks will increase wherever the number of WASH-related deaths increases (as in EAP until the late 1970s, in SA and MIDEAST until the early 1990s, and still today in SSA).

On the other hand, income growth and infrastructure service coverage expansion over time are also associated with lower WASH-related mortality rates, and these relationships reduce the potential remaining economic benefits. The trajectory of the potential economic benefits (aggregated to the country-level) from eliminating mortality attributable to poor water and sanitation thus depends on the relative speed and direction of two pressures: 1) the increasing economic benefits per death avoided and 2) the increasing and then decreasing numbers of WASH-related deaths, which themselves are determined by declining mortality rates and increasing population. In most less-developed countries, the net result of the complex relationship between income, population, and potential economic gains from eliminating WASH-related deaths is that economic benefits initially rise when coverage with improved water and piped services is low, and mortality and population growth are high, then reach a peak, and subsequently decline once population growth slows and mortality rates drop sufficiently to counteract the rising demand (willingness to pay) for mortality risk reductions. In addition, consistent with the countervailing influence of the drivers identified above, the timing and size of the regional maxima in annual potential economic gains from eliminating WASH-related diseases both shift depending on assumptions about the associations between mortality rates, WASH coverage, and income (refer to materials S1 for additional details).

These results may be put in perspective by comparing the potential economic value of health gains from eliminating WASH-related mortality to the total GDP for the regions involved (Figure 5, Panel B). As shown, economic gains as a percentage of GDP steadily decline over time, from over 1% of developing world GDP in 1975, to 0.01% by 2050. This decline occurs because GDP growth from increased population and economic development increases at a faster rate than potential WASH-related gains. Economic gains as a percentage of GDP should not be interpreted as the net economic effect of improved water and piped services, however, because (a) the causal role these services play in reducing disease is uncertain, (b) these services provide other benefits besides mortality reductions, and (c) large expansions of improved water (especially piped services) would cost money, and we have not compared the economic benefits of reduced mortality to the costs of implementing these WASH improvements.

Overall, the net present value of potential future economic gains from 2012–2050, discounted at 3%, are greatest in SSA ($0.5 trillion in the base case, or 0.8% of the net present value of GDP for the same period), reflecting the larger future mortality from WASH-related diseases in that region, followed by South Asia ($0.2 trillion in the base case, or 0.06% of the NPV of GDP). As with the timing and magnitude of the regional peaks in potential economic gains, their overall sum is highly sensitive to model parameters (Table 6).

## Discussion

This study estimated the potential economic gains from eliminating the mortality associated with poor water and sanitation over a long time horizon. The analysis provides a broad perspective on the trajectory of WASH service coverage, WASH-related mortality, and its economic consequences over time, as less-developed countries experience economic and demographic growth.

Our projections of the trends of rising access to water and sanitation services and of declining mortality rates are generally consistent with the cross-country data analyzed in this paper. Our simulated declines in mortality rates are also consistent with evidence that mortality rates from infectious diseases have recently declined across much of the globe [5], [20], [25]. We note that the water and sanitation coverage trends in the JMP data covering the period 1990–2010 are somewhat faster than those that are predicted for the remainder of our simulation. Coverage gains in high-growth regions slow in the future as access approaches 100% (consistent with the leveling off detected in the alternative regression specifications described above). Also, the long-term GDP growth rates of 2–5% used for our past and future estimations are lower than those of several regions during the 1990–2010 period, most notably EAP but also LAC and MIDEAST (which have all seen rapid recent increases in access to piped services). In contrast, coverage increases for piped services in SA and SSA for 1990–2010 are slower than the projections, and projected WASH-related mortality rates will remain well above zero in SSA even in 2050.

To better understand these mortality rate projections, the factors driving the large differences in the WHO estimates of the WASH-related mortality for SSA (higher) and for SA (lower) deserve further research and attention. This divergence may stem from differences in per capita income, access to other health services, greater access to improved (non-piped) water and sanitation, or changing demographics. In fact, our country-level analysis of WASH-related mortality rates suggests that much of the difference between SA and SSA can be explained by the control variables – income, infrastructure coverage, and others – included in the regressions in Table 3, as the SA regional fixed effect is not consistently statistically significant.

Our projections suggest that the potential economic gains from eliminating WASH-related mortality do not always decrease in tandem with economic growth, even as coverage rates increase and WASH-related mortality rates decline. This happens because of the countervailing effects of expansion in services on the one hand, which decreases the potential gains from eliminating WASH-related diseases, and the effects of population and income growth on the other hand, which increase the value of (and demand for) these mortality risk reductions. In 1975, WASH-related mortality rates in less-developed regions were much higher than they are today. Even so, the potential economic gains from reducing WASH mortality in SSA were lower than they are today because the affected population was smaller and incomes (and the corresponding economic value of mortality risk reductions) were also lower. We estimate that regions such as EAP, LAC, MIDEAST, EURCA, and even SA have had a decreasing economic burden of WASH-related mortality since reaching a peak in the late 1970s (EAP) and early 1990s (LAC, MIDEAST, EURCA and SA), even without achieving universal coverage with piped services. Similar to SSA today, those regions experienced increases in potential economic benefits from reduced WASH-mortality leading up to this peak, when their population growth rates were higher, and before mortality rates dropped to low levels.

In SSA, we estimate that the economic gains from eliminating WASH-related mortality are still increasing. In other words, SSA is still on the rising portion of the inverted U-shaped curves simulated in this paper because of the region's high population growth, high mortality, and low current levels of coverage with improved water and sanitation services. Through sensitivity analysis, we determined that the rising trends in both WASH-related deaths and these potential economic gains in SSA are relatively robust to changes in the assumptions of the simulation model, though the timing of the peak varies substantially. Only under the most optimistic assumptions about income growth (5–10% per year), and strong associations between coverage, mortality and income (Table 4), do our simulations show that this rising trend is now near its peak. In SA, average incomes are higher, and overall potential economic gains from increased water and sanitation coverage are slowly declining, but average statistics mask the reality that hundreds of millions of very poor people, for example those living in the Ganges Plain, earn less than $2 per day. As in SSA, the demand for mortality risk reductions in these locations likely remains low [9], [26], and the economic benefits of expanding coverage and reducing WASH-related diseases may continue to rise for some time, especially given high rates of population growth.

These findings suggest that the economic burden of WASH-related disease in SSA is likely to remain high without additional investments in water and health related infrastructure by less-developed country governments and the international community. In fact, rising demand for the economic benefits of WASH improvements – whether reduced WASH-related illness and mortality or simply the greater convenience these services provide – that accompanies economic development is probably one of the most important factors that creates societal pressure for governments to invest in capital-intensive piped services. People in less-developed nations generally want access to piped water and sanitation, and their ability to pay for these improvements increases sharply during the transition to middle-income status. As a variety of investments are made, the rise in economic gains from further disease burden reductions slows and eventually begins to decline. For example, the recent expansion in piped services in China did not occur by chance: it accompanied strong government investment programs in public infrastructure, double-digit income growth, and greater household demand for such services [27]. Wherever economic development is successful and broadly based, and governments are responsive to the demands of citizens, the problem of WASH-related mortality is likely to be reduced substantially without new technological breakthroughs.

Still, conventional piped services are expensive and impose large real resource costs on poor countries. Piped technologies will not be the best solution in all places and at all times. In EAP, improved water and piped service access rose to 70% and 50%, respectively, before the potential economic gains from further WASH-related mortality risk reductions declined to low levels. The cost of expanding piped water and sewerage alone to comparable levels in lagging regions such as SA and SSA will be substantial, totaling about $70 billion dollars per year, equivalent to 1 and 2% of the GDP of these respective regions. This estimate was obtained by noting that 2 billion people (out of 2.2 billion total) did not have piped water and sanitation in these regions in 2010. To raise coverage levels to 40–50%, roughly 1 billion people (or 200 million households) would need to gain access. Assuming that the full economic cost of piped water and sanitation per household is $30 per month [9], the annual costs of covering these people would be about $72 billion.).

We caution that the estimates of economic gains in this paper do not provide sufficient information for a full economic assessment of the costs and benefits of expanding water and sanitation coverage because (a) the relative costs of different types of infrastructure have not been discussed; (b) our analysis only values mortality benefits (though regression results with DALYs suggest that the associations for overall disease burden including morbidity are similar to those for mortality, see Table S7); and (c) our cross-country regression models are estimated using a short time series and are insufficient for determining the causal impact of water and sanitation on mortality. Simply financing an expansion of piped services is not sufficient; success requires consistent operation and maintenance efforts and capital reinvestment, as well as repayment of debt. If the uptake of non-piped solutions that provide substantial health benefits were subject to the same demand-side forces that push people with rising incomes to invest in piped services, then mortality declines could accelerate independently of large-scale and costly initiatives to expand piped water and sewerage. However, empirical evidence suggests that the demand for cheap, decentralized interventions in the WASH sector is very low, possibly because non-piped services do not bring many of the other benefits that many households value [9], [18], [28], [29], [30]. This suggests that sustaining widespread use of non-piped, low-cost technologies will remain a major challenge.

We also note that there are important data limitations in this analysis, and we especially warn against drawing any conclusions regarding a causal dynamic in the relationships between income, coverage with improved water and sanitation services, and reduced mortality from WASH-related diseases. First, data constraints and measurement problems severely limit attempts to make accurate projections of the potential economic gains from eliminating WASH-related illnesses. The regression estimates used to parameterize the model rely on incomplete country-level data (roughly one third (n = 45) of the 140 less-developed countries included in the final simulation model lacked sufficient data on control variables for inclusion in the regression models), though mortality data were more complete) which also obscure important sub-national differences in income, coverage, and mortality. Uncertainties associated with the burden of disease data available from the WHO are large. Second, our predictions could be inaccurate if significant changes occur in the current trajectories of economic development (e.g., accelerated growth) and WASH improvements (e.g., significantly enhanced interventions or innovations), or if structural changes in the simulated dynamics occur. As discussed above, the precise timing and magnitude of the regional peaks in potential economic gains are very sensitive to model assumptions, and the outlier case of Equatorial Guinea highlights that the links we detect between income, infrastructure coverage and WASH-related mortality are not inevitable. The fact that we do not find statistically significant associations between improved sanitation and WASH-mortality at the country level is also surprising, given the literature on the impacts of sanitation [7], [8].

Similarly, this paper does not explicitly consider the role of hygiene, which is also probably correlated with but not determined by infrastructure [31]. It is also important to note that our projections are for WASH-related mortality only, which as defined only covers infectious diseases. Countries that seem to be doing well in these terms (such as China) still face severe water supply challenges, for example related to industrial pollution, climate change, or water scarcity. Nonetheless we believe that the basic insights provided by these projections are useful for thinking about the health and livelihoods implications of WASH-related diseases in less-developed countries.

## Supporting Information

Figure S1 **Scatter plots of VSL estimates published since around 2000 for industrialized and middle income** **countries, with low and high income elasticity curves superimposed (Note: Panel B shows the data on** **a log scale).**
(TIF)

Figure S2 **Hybrid S-shaped VSL curve used in the simulation model, combining an exponential function fit to empirical VSL estimates and an income elasticity of 0.55 at higher incomes.**
(TIFF)

Figure S3 **Tornado charts showing sensitivity of model outcomes to assumptions (base case outcome shown by** **vertical red line) for A) coverage with improved water; B) coverage with piped services; C) average** **projected WASH-mortality rate in 2050; D) value of projected potential health gains from eliminating** **WASH-related illnesses in developing countries in 2050; and E) present value of projected potential** **health gains from eliminating WASH-related illnesses in developing countries from the present to** **2050.**
(TIF)

Materials S1 **The long-term dynamics of mortality benefits from improved water and sanitation in less developed** **countries.**
(DOC)

Table S1 **Summary of international studies reviewed for this paper.**
(DOCX)

Table S2 **Region groupings of countries in our analysis.**
(DOCX)

Table S3 **Estimation of population coverage with piped water.**
(DOCX)

Table S4 **Estimation of population coverage with all improved water.**
(DOCX)

Table S5 **Estimation of population coverage with all improved sanitation.**
(DOCX)

Table S6 **Estimation of WASH-related mortality (deaths per thousand people per year); alternative random** **effects model specifications.^a^**
(DOCX)

Table S7 **Estimation of DALYs due to WASH-related illnesses (DALYs per thousand people).^a^**
(DOCX)
