# Supplementary figures and images for: The Long-Term Dynamics of Mortality Benefits from Improved Water and Sanitation in Less Developed Countries
Source: PLoS One. 2013 Oct 8;8(10):e74804. doi: 10.1371/journal.pone.0074804 (PMC3792953; doi:10.1371/journal.pone.0074804)

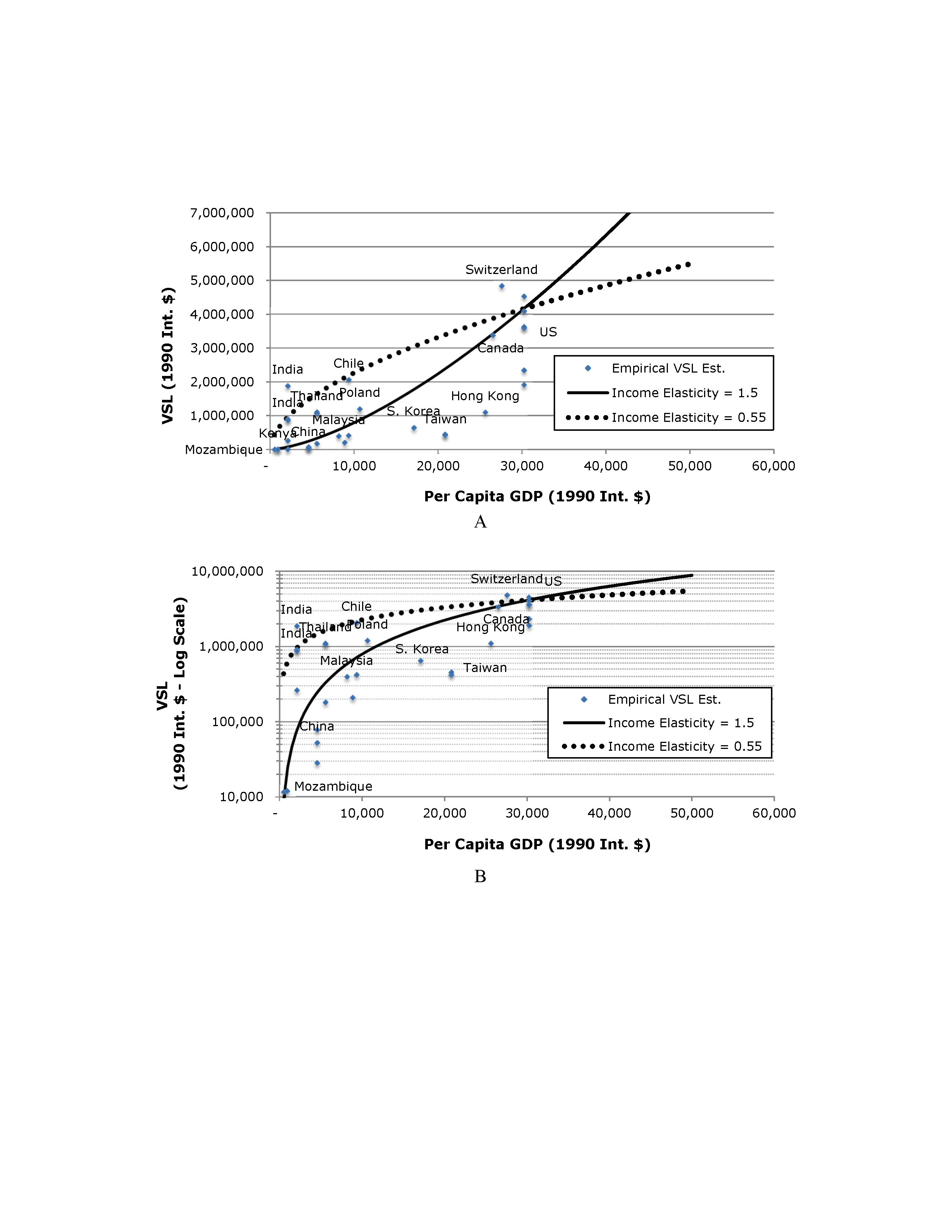

Supplement: Figure S1 — Scatter plots of VSL estimates published since around 2000 for industrialized and middle income countries, with low and high income elasticity curves superimposed (Note: Panel B shows the data on a log scale). (TIF) [file pone.0074804.s001.tif]

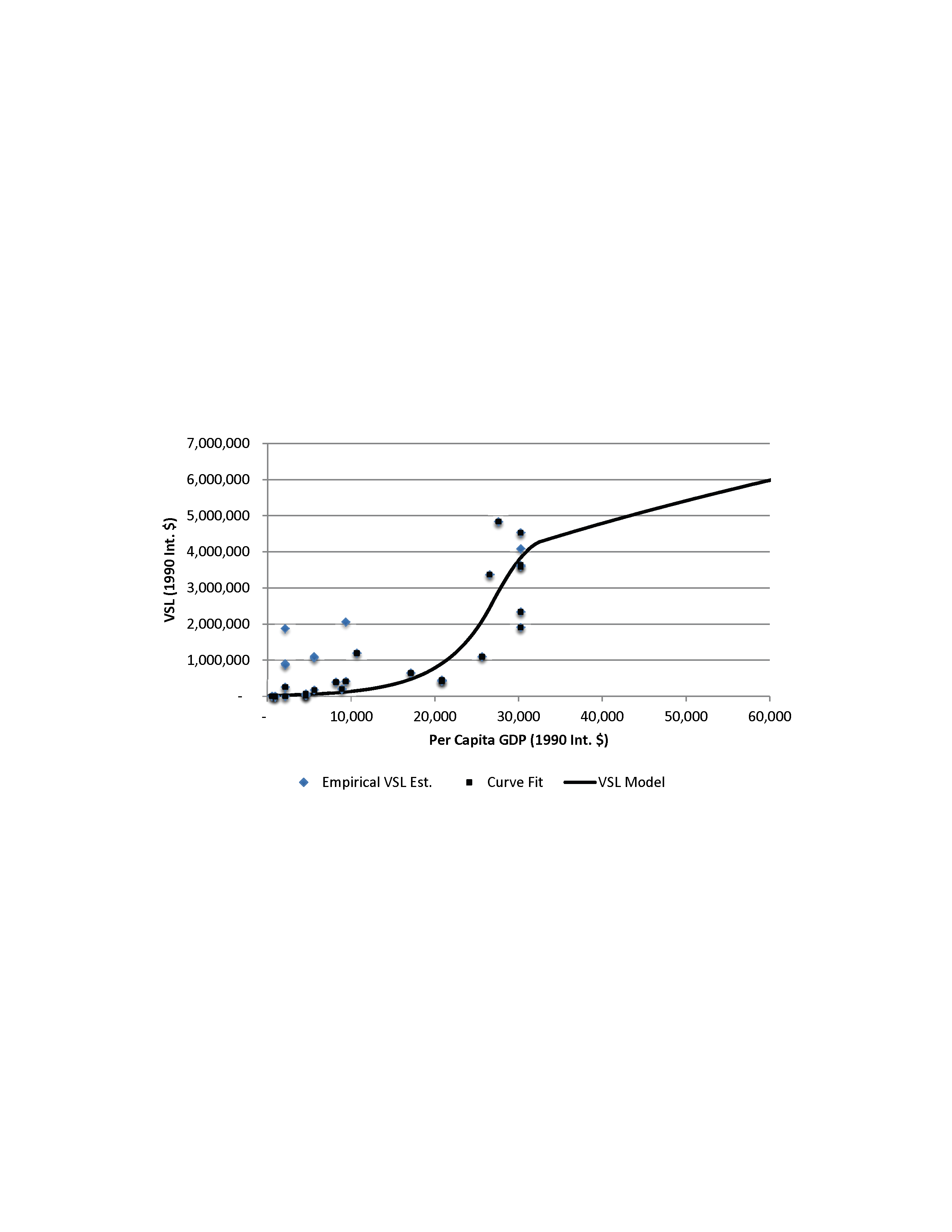

Supplement: Figure S2 — Hybrid S-shaped VSL curve used in the simulation model, combining an exponential function fit to empirical VSL estimates and an income elasticity of 0.55 at higher incomes. (TIFF) [file pone.0074804.s002.tif]

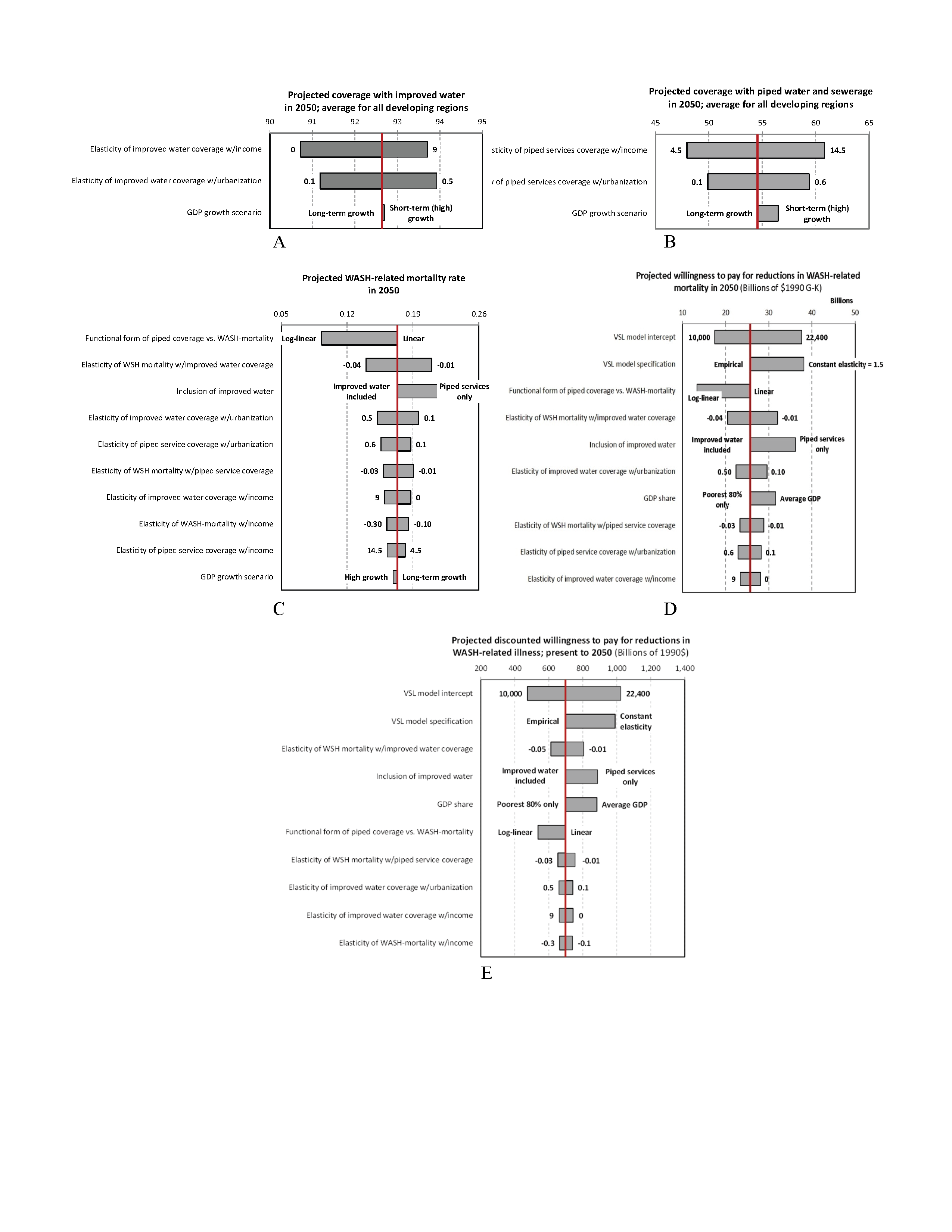

Supplement: Figure S3 — Tornado charts showing sensitivity of model outcomes to assumptions (base case outcome shown by vertical red line) for A) coverage with improved water; B) coverage with piped services; C) average projected WASH-mortality rate in 2050; D) value of projected potential health gains from eliminating WASH-related illnesses in developing countries in 2050; and E) present value of projected potential health gains from eliminating WASH-related illnesses in developing countries from the present to 2050. (TIF) [file pone.0074804.s003.tif]
