# Supplementary material for: The Long-Term Dynamics of Mortality Benefits from Improved Water and Sanitation in Less Developed Countries
Source: PLoS One. 2013 Oct 8;8(10):e74804. doi: 10.1371/journal.pone.0074804 (PMC3792953; doi:10.1371/journal.pone.0074804)
Supplement: Materials S1 — The long-term dynamics of mortality benefits from improved water and sanitation in less developed countries. (DOC) [file pone.0074804.s004.doc]

**Materials S1.** The long-term dynamics of mortality benefits from improved water and sanitation in less developed countries.

This supplementary appendix provides additional details on our approach for estimating, parameterizing, and simulating the equations presented in the Methods section. In the first section, we describe the construction of the WASH-related mortality and the value of statistical life (VSL) variables, and offer additional information on the model specifications and data used in the cross-country regression analysis. In part 2 (Additional regression results), we present alternative specifications developed to assess the sensitivity of the results presented in Tables 2 and 3 of the main paper. Finally, in part 3 (Sensitivity analysis) we present more detailed sensitivity analyses of the results generated by our simulation model.

Modeling

*WASH-related mortality variable*

The literature provides limited insight on the relationships between WASH-related disease incidence and case fatality rates, income, and access to various levels of improved water and sanitation. Few reliable measures of incidence of and mortality from WASH-related diseases in different countries exist. (In the description that follows, we use the term mortality attributable to WASH to mean the ratio of the total number of deaths due to WASH to the total number deaths. The terms WASH-related death rate and WASH-related mortality refer to the ratio of the total number of deaths due to WASH to the population in a country.). The basic methodology used to calculate the mortality attributable to WASH is described in Fewtrell et al. [[1]](#footnote-2), but this includes: a) infectious diarrhea (90% of deaths), which is determined on the basis of access to safe water and sanitation; b) malnutrition, 39-61% of which is estimated to be attributable to inadequate WASH and its consequences (based on expert opinion); and c) intestinal nematode infections, schistosomiasis, trachoma and lymphatic filariasis, 66% of which is attributed to poor WASH . For malnutrition, we use the more conservative estimate of 39% in all countries, given that country-specific rates have not been published.

We convert country estimates of the mortality attributable to WASH as a percentage of all deaths into a WASH-related death rate by multiplying this percentage by the total number of deaths and dividing by the total population of each country that is represented in the data:

$d_{it}^{WSH}=\sum_{j} pd_{ijt}^{WSH}\cdot\frac{Deaths_{ijt}}{Pop_{it}}$, (Eq. S1)

where is the death rate that is attributable to poor water, sanitation and hygiene; is the mortality from condition *j* that is attributable to WASH, as specified in the percentages listed above; and and are the total number of deaths from condition *j* and population, all for country *i* in year *t*. In the sample of developing countries for 2002-2008, these death rates range from a low value of 0 in several countries to roughly 5.5 deaths per thousand people per year in Angola in 2004, with a mean value of 0.51 deaths per thousand.

*Value of a statistical life (VSL) variable*

We specify the VSL (in 1990 International Geary-Khamis dollars) in our model to be dependent on income, following the evidence on VSLs from meta-analyses in the literature [3-5]. The majority of studies that seek to value mortality risk reduction have been conducted in industrialized countries and in particular in the US and Canada. One recent review of international VSL studies lists 30 studies conducted in 12 countries , none of which were from less-developed countries; another examines 50 studies (27 in the US), only three of which were in non-industrialized countries (and all in India) . We therefore use an approach that incorporates the findings from both (1) these meta-analyses on the relationship between income and VSL in industrialized countries; and (2) the limited empirical data on the VSL for less-developed countries, summarized in Table S1 below. Figure S1 (Panels A and B) provides a scatter plot of these published VSL estimates expressed in 1990 international dollars, superimposed with “high” and “low” income elasticity curves proposed by other authors .[[2]](#footnote-3)

As shown, the estimates derived using an income elasticity of the VSL of 1.5 are generally consistent with the lower end of estimates for developing countries found in the literature up to a per capita GDP of about 10,000 USD.[[3]](#footnote-4) This elasticity is much higher than the 0.5-0.6 income elasticity found in developed world studies . Thus, in order to preserve the relatively well-established VSL-income relationship for rich countries while acknowledging the empirical evidence from developing countries, we use a functional relationship that does not depend on constant income elasticities. Examining the published estimates, it appears that VSLs are initially very low, before rising steeply with incomes between 20,000-30,000 USD, and then leveling off again with lower income elasticities of 0.5-0.6. To accommodate this S-curve relationship, we fit an exponential function to the low estimates for developing countries up to the end of this transition zone (approximately $30,000), and follow the results from the meta-analysis literature from industrialized countries thereafter, assuming that the income elasticity of the VSL is 0.55 (Figure S2).[[4]](#footnote-5) In the sensitivity analysis for our paper, we use a relationship with a constant elasticity over all income levels at the central estimate of 1.5 used by Hammitt and Robinson (2011), and we also test the implications of varying this elasticity over a range of 1 to 2. Although this S-shaped curve is admittedly ad hoc, we believe that it is the best characterization of the VSL estimates available in the published literature.

*Alternative Regression model specifications*

The paper presents results from several basic specifications of the cross-country regressions described by equations 1 and 2. In this appendix, we also explore the following alternative model specifications:

1. Equation 1 (Infrastructure coverage): Alternative specifications with piped water;
2. Equation 1 (Infrastructure coverage): Improved water only, Improved sanitation only;
3. Equation 2 (WASH-related mortality): Alternative specifications for WASH-related mortality; and
4. Equation 2 (DALYs): Replacing the outcome of WASH-related mortality with WASH-related disability-adjusted-life-years (which include morbidity).

*Data*

This section describes the sources for the control variables included in the cross-country regression models:

1. **Water and sanitation infrastructure coverage.** Data on coverage with improved water and sanitation services for the period 1990–2010 were obtained from the Joint Monitoring Program (JMP). Improved water includes plot or yard tap, public tap/standpipe, tube well/borehole, protected dug well, protected spring, rainwater collection, and piped water; improved sanitation includes flush or pour-flush to piped sewer system, septic tank or pit latrine, ventilated improved pit latrine, pit latrine with slab, composting toilet, and sewerage.
2. **WASH-related mortality**. As discussed in the main paper, we utilize country-level data from the WHO’s Environmental Burden of Disease project for 2002 and 2008.
3. **Income**. The Groningen Growth and Development Center (GGDC) database was used for per capita GDP (all normalized to 1990 Geary-Khamis (G-K) dollars).
4. **Urban population**. Percentage of total population in a country that is located in an urban area. This variable was created by dividing urban population by total population, both of which were reported by the WHO/UNICEF JMP. Urban population projections for the future were obtained from: <http://esa.un.org/unpd/wup/index.htm>. We expect that higher levels of urbanization will be associated with higher piped water coverage.
5. **World region dummy variables**. We generated dummy variables for the major world regions in order to control for regional differences that might influence piped water coverage and WASH-related death rates. We use the following country groupings: 1) Sub-Saharan Africa (SSA); 2) Latin America and the Caribbean (LAC); 3) Middle East (MIDEAST); 4) Eastern Europe (EURCA); 5) South Asia (SA); and 6) East Asia and the Pacific (EAP). The full list of countries in each of these regions is shown in Table S2.
6. **Inequality**. We obtained the percentage of national GDP for the lowest 80% of the income distribution from the World Bank database, as a measure of inequality. Higher percentages imply greater equality.
7. **Fertility**. Fertility rates were obtained from the United Nations. Only year 2008 rates are available. Because fertility rates are highly endogenous, we included this variable mainly to test whether the associations of greatest interest to us (specifically the effect of improved water services on mortality rates) are robust to model specifications that include it.
8. **Literacy**. Adult literacy rates for 2005 were also obtained the United Nations. We expect that death rates will be negatively correlated with literacy rates.
9. **Child vaccination rates.** We included the WHO-recorded immunization rates for diphtheria, tetanus toxoid, and pertussis (DTP3) among 1-year-olds (%) as a proxy for the quality of health services provision. We expect that access to health services is negatively associated with mortality due to diarrheal disease.
10. **Governance variables**. Obtained from the Center for Systemic Peace’s Integrated Network for Societal Conflict Research (INSCR). We expect that higher coverage levels with infrastructure and lower deaths will be associated with positive governance and stability measures.

1. **Democracy-Autocracy score**: This is a governance measure that seeks to account for the extent of democracy and autocracy in a country. The variable is derived by subtracting a country’s autocracy score (-10 to 0) from its democracy score (0 to 10). A Democracy-Autocracy score of +10 indicates the most strongly democratic country possible; -10 is the most autocratic.

2. **Regime Durability**. This variable indicates the number of years since the most recent regime change. A regime change is defined by a three-point change in the Democracy-Autocracy score over a period of three years or less.

3. **Coups d’Etat**. We used the coup indicator to create a dummy variable indicating a successful coup in the last five years.

1. **Bilateral aid for WASH**. Data on total aid received for WASH by country was obtained from the OECD for many countries after 1996, in three categories: aid for large systems (water treatment plants and networks), aid for basic systems, and water resources planning and management. As far as we know, these are the only comprehensive data available for WASH aid.
2. **Linear time trend and/or year dummy variables**. For the piped water coverage model, we defined a linear time trend variable as equal to the year of the observation minus 1990, which was the first year in our dataset. We also created dummy variables for each year to control for non-linear effects over time, and created a set of time-region interactions as additional controls for regionally-differentiated time effects.

Additional regression results

*Equation 1 (Infrastructure coverage): Alternative specifications with piped water*

Regression results for alternative specifications of equation 1 with piped water are reported in Table S3. The first alternative specification controls for international aid flows to the WASH sector, and reduces the sample size to fewer than 150 observations and three years of panel data. In this specification, the random effects coefficient estimates are unaffected, but the fixed effects model becomes unstable (possibly due to restriction of the length and number of observations in the panel).

We also estimated several additional models in order to assess the sensitivity of the model results to assumptions about functional form (linear and squared terms) and coverage variables. The model fit for the models with linear per capita GDP was generally not as good as the simple log GDP model, and adding squared log GDP terms only provided small improvements in model fit. In these models, the squared log GDP term was consistently statistically significant and negative, and the linear terms for income and urbanization were slightly larger for the random effects specification than in the fixed effects specification (consistent with the simple log specifications presented in the main paper). We interpret this as evidence that a diminishing rate of expansion of piped services may apply late in the development path of countries, once they approach full coverage.

*Equation 1 (Infrastructure coverage): Improved water only*

Regression results for equation 1 with improved water are reported in Table S4. We find that per capita income is less strongly correlated with improved water coverage than with piped water. In addition, particularly in the fixed effects model, the urban population variable was less significant than in the model with piped water (Table 2 in the main paper). Increased inequality has a negative association with coverage (significance at the 5 and 10% level). Other results are similar to those for piped water: the governance variables are only weakly related to coverage (democracy has a positive association and coups have a negative relationship with coverage), and there is a positive trend in coverage over time.

*Equation 1 (Infrastructure coverage): Improved sanitation only*

Regression results for equation 1 with improved sanitation are reported in Table S5. As with the other water-sanitation infrastructure categories, per capita income is strongly correlated with coverage, particularly in the sample of less developed countries. Urbanization is again positively related to coverage, and there is increasing coverage over time. As with the other levels of infrastructure, the governance variables do not appear strongly related to improved sanitation coverage.

*Equation 2 (WASH-related mortality): Alternative specifications for WASH-related mortality*

The results of alternative specifications for the regressions of WASH-related death rates (equation 2) are presented in Table S6. We find some evidence that the associations between coverage variables and death rates may be nonlinear, based on alternative specifications that include higher order squared terms, or log coverage terms. The basic model that includes only linear terms is shown in Column 1. Column 2 then presents a model with log coverage terms; in this specification, a 1-log increase in % coverage with piped water is associated with a decline in WASH mortality of 0.38 deaths per thousand, whereas the effect of a 1-log increase in % coverage with other improved water is smaller (decline of 0.24 deaths per thousand). In addition, when higher order terms are included alongside the linear coverage terms in the model, the coefficient for the linear piped water coverage increases by a factor of two (to -0.4), and the squared piped water coverage is positive and significant (Column 3); while the terms for coverage with other improved water sources are not significant. Finally, the most parsimonious model that has the best fit contains a log coverage term for piped water and a linear term for improved water (Column 4). Taken together, these alternative specifications suggest that there is: 1) a concave relationship between death rates and piped water coverage, whereby declines in WASH-related deaths decrease as coverage increases, and 2) a linear relationship between declines in WASH-related deaths and coverage with other improved water sources. Improved sanitation is not significantly associated with the WASH-related death rate in any of these models, while the association between mortality and log income per capita remains statistically significant and stable (a 1-log increase in income is associated with a decline in WASH-mortality of 0.18-0.23 deaths per thousand). Similar tests on inclusion of different income terms reveal that the log income specification outperforms models with linear or higher order income terms, and that model fit declines with models with shorter lags (1 and 3 years).

*Equation 2 with DALY outcome*

Results using DALY outcomes rather than WASH-related mortality are generally similar to those described above (Table S7). However, results from the fixed effects specification with this outcome are unstable, probably due to the short duration of the panel and to the issues described in the main text (regarding measurement error in the disease burden calculations). In the random effects models, a 1% increase in improved or piped water coverage is associated with roughly -0.6 fewer DALYs per thousand people per year, and a 1 log increase in income is associated with 8 to 13 fewer diarrheal disease DALYs per thousand people. In these models, as in the mortality models, improved sanitation coverage is not significantly associated with health outcomes.

Sensitivity analyses for simulated outcomes

The bounds of the results from sensitivity analyses reported in the main paper utilize the lower and upper bound estimates of the parameters summarized in Table 4, which are based on the 90% confidence intervals from the random and fixed effects model estimates for piped and other improved water coverage (Tables 2 and S4), and the random effects regression model estimates for coverage and WASH-mortality (Table 3). Also included in the construction of low and high estimates of outcomes are different assumptions about economic growth (long term or short term), the VSL (empirical curve described above or constant-elasticity VSL suggested by Hammitt et al. [4]), and the relevant income level for calculation of country-level VSLs (average per capita GDP or average per capita GDP to the lowest 80%). In this section, we present tornado charts that provide additional details on the varying importance of these parameters in affecting the following simulated outcomes:

1. Coverage with improved water;
2. Coverage with piped water and sewerage;
3. Average projected developing country WASH-mortality rate in 2050;
4. Value of projected potential health gains from eliminating WASH-related illnesses in developing countries in 2050; and
5. Present value of projected potential health gains from eliminating WASH-related illnesses in developing countries from the present to 2050.

As detailed in Figure S3, future coverage projections are most sensitive to assumed associations between coverage and GDP growth and urbanization, and are somewhat less sensitive to the assumed GDP growth scenario. Mortality rates are most sensitive to: 1) the functional form of the relationship between coverage with piped services and mortality rates (assuming that mortality declines with the log of coverage rather linearly leads to much lower projections); and 2) the strength of the association between WASH-related mortality and coverage with improved water. Other less important parameters driving uncertainty in these projections are associations between income, urbanization, and coverage with piped services and improved water, and the association between mortality rates and piped services. Finally, the most critical factors for varying the estimates of economic benefits from reducing WASH-related illnesses are the parameters that determine the relationship between the value of mortality risk reductions and income, the magnitude of gains from reducing morbidity from WASH-related diseases relative to mortality, and the degree of association between the mortality rate and coverage with improved water, and similar parameters drive variation in the estimated willingness-to-pay for avoiding WASH-illnesses in 2050.

The timing and size of the regional maxima in potential economic gains from eliminating WASH-related diseases also shifts considerably depending on assumptions about the associations between mortality rates, WASH coverage, and income. In general, lower associations between income and urbanization and our model outcomes (coverage and mortality rates) tend to both increase the size of the peak in potential gains and push it farther into the future.

References

Table S1 References

1. Baranzini, Andrea; Ferro Luzzi, Giovanni. 2001. The Economic Value of Risks to Life: Evidence from the Swiss Labour Market. *Swiss Journal of Economics and Statistics* 137(2): 149-70.
2. Bhattacharya, S, A Alberini, and M L Cropper. 2007. The value of mortality risk reductions in Delhi, India. *Journal of Risk and Uncertainty* 34 (1): 21-47.
3. Blomquist, G.C., Miller, T.R., Levy, D.T., 1996. Values of risk reduction implied by motorist use of protection equipment: new evidence from different populations. *Journal of Transport Economics and Policy* 30: 55–66.
4. Dreyfus, M.K. and W.K. Viscusi. 1995. Rates of Time Preference and Consumer Valuations of Automobile Safety and Fuel Efficiency. *Journal of Law and Economics* 38 (1): 79-105.
5. Gayer, T., J.T. Hamilton, and W.K. Viscusi. (2000). Private Values of Risk Tradeoffs at Superfund Sites: Housing Market Evidence on Learning About Risk. *Review of Economics and Statistics* 82 (3): 439-45.
6. Gibson, J, S Barns, M Cameron, S Lim, F Scrimgeour, and J Tressler. 2007. The value of statistical life and the economics of landmine clearance in developing countries. *World Development* 35 (3): 512-531.
7. Giergiczny, M. 2008. Value of a Statistical Life –– The Case of Poland. *Environmental and Resource Economics* 41: 209-221.
8. Guo, X. and J.K. Hammitt. 2009. Compensating Wage Differentials with Unemployment: Evidence from China. *Environmental and Resource Economics* 42 (2): 187-209.
9. Hammitt, J K and M E Ibarrarán. 2006. The economic value of fatal and non-fatal occupational risks in Mexico City using actuarial-and perceived-risk estimates. *Health Economics* 15 (12): 1329-1335.
10. Hammitt, J K and Y Zhou. 2006. The economic value of air-pollution-related health risks in china: A contingent valuation study. *Environmental and Resource Economics* 33 (3): 399-423.
11. Jenkins, R., N. Owens, and L. Bembenek Wiggins. 2001. Valuing Reduced Risks to Children: The Case of Bicycle Safety Helmets. *Contemporary Economic Policy* 19 (4): 397-408.
12. Jeuland, M.; M. Lucas; J. Clemens; D. Whittington (2009). A Cost Benefit Analysis of Vaccination Programs in Beira, Mozambique. *World Bank Economic Review* 23 (2): 235-267.
13. Kim, S.W. and P.V. Fishback. 1999. “The Impact of Institutional Change on Compensating Wage Differentials for Accident Risk: South Korea, 1984-1990. *Journal of Risk and Uncertainty* 18 (3): 231-248.
14. Kremer, M, J Leino, E Miguel, and A Zwane. 2009. *Spring Cleaning: Rural Water Impacts, Valuation and Property Rights Institutions.* National Bureau of Economic Research Cambridge, Mass., USA.
15. Liu, J T, J K Hammitt, and J L Liu. 1997. Estimated hedonic wage function and value of life in a developing country. *Economics Letters* 57 (3): 353-358.
16. Liu, J-T, and J.K. Hammitt. 1999. “Perceived Risk and the Value of Workplace Safety in a Developing Country.” *Journal of Risk Research* 2 (3): 263–75.
17. Lott, J.R. and R.L. Manning. 2000. Have Changing Liability Rules Compensated Workers Twice for Occupational Hazards? Earnings Premiums and Cancer Risks. *Journal of Legal Studies* 29: 99-130.
18. Maskery, B., Z. Islam, J. Deen, and D. Whittington. 2008. “An Estimate of the Economic Value That Parents in Rural Bangladesh Place on Ex-ante Mortality Risk Reductions for Their Children.” Working Paper. Department of Environmental Sciences and Engineering, University of North Carolina, Chapel Hill.
19. Melhuish, C., A. Ross, M. Goodge, K.K.C. Mani, M.F.M. Yusoff, and R. Umar. 2005. *Accident Costing Report AC5: Malaysia.* Asian Development Bank, Association of Southeast Asian Nations, Regional Road Safety Program.
20. Meng, R. and D.A. Smith. (1999). The Impact of Workers' Compensation on Wage Premiums for Job Hazards. *Applied Economics* 31(9), 1101-1108.
21. Ortuzar, J.D., L.A. Cifuentes and H.C.W.L. Williams. 2000. Application of Willingness-to-Pay Methods to Value Transport Externalities in Less Developed Countries. *Environment and Planning A* 32 (11): 2007-2018.
22. Shanmugam, K.R. 1997. Compensating Wage Differentials for Work Related Fatal and Injury Accidents. *The Indian Journal of Labour Economic* 40 (2).
23. Shanmugam, K R. 2000. Valuations of life and injury risks. *Environmental and Resource Economics* 16 (4): 379-389.
24. Shanmugam, K R. 2001. Self selection bias in the estimates of compensating differentials for job risks in India. *Journal of Risk and Uncertainty* 22 (3): 263-275.
25. Siebert, W.S. and X. Wei. 1994. Compensating Wage Differentials for Workplace Accidents: Evidence for Union and Nonunion Workers in the UK. *Journal of Risk and Uncertainty* 9 (1), 61-76.
26. Vassanadumrongdee, S and S Matsuoka. 2005. Risk perceptions and value of a statistical life for air pollution and traffic accidents: Evidence from Bangkok, Thailand. *Journal of Risk and Uncertainty* 30 (3): 261-287.
27. Wang, H and J Mullahy. 2006. Willingness to pay for reducing fatal risk by improving air quality: A contingent valuation study in Chongqing, China. *Science of the Total Environment* 367 (1): 50-57.

1. Available at: <http://www.who.int/quantifying_ehimpacts/national/countryprofile/intro/en/index.html> (Accessed October 2010). The data are available for 2002, 2004 and 2008. [↑](#footnote-ref-2)
2. These GDPs are in international dollars as reported in the Penn World Tables for 2007, deflated to 1990, for consistency with the main article. [↑](#footnote-ref-3)
3. We do not believe that the high VSL estimates for India, Thailand and Chile (that appear considerably larger than suggested by this curve) are representative of developing country VSLs because they were conducted in urban settings with semi-skilled or skilled workers, or among individuals owning motor vehicles. [↑](#footnote-ref-4)
4. Five studies were excluded from the curve estimation. Four of the studies (Shanmugam 2001, Shanmugam 2000, Shanmugam 1997, Vassanandum and Matsuoko 2005) were conducted in urban areas among a relatively well-off segment of the population. Ortuaz et al (2000) presents two estimates of the VSL, one of which is 500 percent larger than the base estimate, and which is also excluded. [↑](#footnote-ref-5)
