# Supplementary material for: The Long-Term Dynamics of Mortality Benefits from Improved Water and Sanitation in Less Developed Countries
Source: PLoS One. 2013 Oct 8;8(10):e74804. doi: 10.1371/journal.pone.0074804 (PMC3792953; doi:10.1371/journal.pone.0074804)
Supplement: Table S1 — Summary of international studies reviewed for this paper. (DOCX) [file pone.0074804.s005.docx]

**Table S1**. Summary of international studies reviewed for this paper.

| **Study** | **Type^a^** | **Country** | **Per Capita GDP** | **Reported VSL** |
| --- | --- | --- | --- | --- |
| ***Developing Countries*** |  |  |  |  |
| Jeuland et al. (2009) | CV/SP | Beira, Mozambique | 504 | 11,700 |
| Kremer et al. (2009) | RP | Kenya (Rural) | 892 | 500 |
| Maskery et al. (2008) | CV/SP | Bangladesh (Rural) | 896 | 12,075 |
| Simon, Cropper, Alberini and Arora (1999) | WR | India | 2,084 | 263,575 |
| Shanmugam (2000) | WR | India (Chennai) | 2,084 | 910,000 |
| Shanmugam (2001) | WR | India (Chennai) | 2,084 | 1,885,000 |
| Bhattacharya, Abernini, and Cropper (2007) | CV/SP | India (Delhi) | 2,084 | 9,068 |
| Shanmugam (1997) | WR | India (Chennai) | 2,084 | 877,500 |
| Guo and Hammitt (2009) | WR | China (Urban) | 4,547 | 52,650 |
| Hammitt and Zhou (2006) | CV/SP | China (Urban & Rural) | 4,547 | 78,163 |
| Wang and Mullahy (2006) | CV/SP | China (Chonging) | 4,547 | 28,470 |
| Vassanandumrongdee and Matsuoko (2005) | CV/SP | Thailand (Bangkok) | 5,558 | 1,072,500 |
| Vassanandumrongdee and Matsuoko (2005) | CV/SP | Thailand (Bangkok) | 5,558 | 1,105,000 |
| Gibson et al. (2007) | CV/SP | Thailand (Rural) | 5,558 | 182,000 |
| Melhuish, Ross, Goodge et al (2005) | CV/SP | Malaysia | 8,154 | 397,800 |
| Hammitt and Ibarraran (2006) | WR | Mexico City | 8,857 | 209,950 |
| Ortuaz, Cifuentes, Williams (2000) | CV/SP | Chile (Santiago) | 9,329 | 2,067,000 |
| Ortuaz, Cifuentes, Williams (2000) | CV/SP | Chile (Santiago) | 9,329 | 421,850 |
| Giergiczny (2008) | WR | Poland | 10,644 | 1,202,500 |
| Kim and Fishback (1999) | WR | South Korea | 17,098 | 650,000 |
| Liu, Hammitt, Liu (1997) | WR | Taiwan | 20,811 | 422,500 |
| Liu and Hammitt (1999) | WR | Taiwan | 20,811 | 455,000 |
| Sibert and Wei (1998) | WR | Hong Kong | 25,600 | 1,105,000 |
| ***Industrialized Countries*** |  |  |  |  |
| Meng and Smith (1999) | WR | Canada | 26,505 | 3,380,000 |
| Baranzini and Ferro Luzzi (2001) | WR | Switzerland | 27,571 | 4,842,500 |
| Lott and Manning (2000) | WR | US | 30,225 | 2,346,570 |
| Dreyfus and Viscusi (1995) | RP | US | 30,225 | 3,598,075 |
| Blomquist et al (1996) | RP | US | 30,225 | 4,536,703 |
| Gayer et al (2000) | RP | US | 30,225 | 3,637,184 |
| Jenkins et al (2001) | RP | US | 30,225 | 1,916,366 |

^a^ CV/SP refers to contingent valuation/stated preference; RP refers to revealed preference; and WR refers to wage-risk.
