# Supplementary material for: The Long-Term Dynamics of Mortality Benefits from Improved Water and Sanitation in Less Developed Countries
Source: PLoS One. 2013 Oct 8;8(10):e74804. doi: 10.1371/journal.pone.0074804 (PMC3792953; doi:10.1371/journal.pone.0074804)
Supplement: Table S2 — Region groupings of countries in our analysis. (DOCX) [file pone.0074804.s006.docx]

**Table S2.** Region groupings of countries in our analysis.

| Region | Countries included |
| --- | --- |
| Developed countries  (DEV_ECON) | Australia, Austria, Belgium, Canada, Cyprus, Denmark, Finland, France, Germany, Greece, Iceland, Ireland, Italy, Japan, Luxembourg, Malta, Netherlands, New Zealand, Norway, Portugal, Singapore, Spain, Sweden, Switzerland, United Kingdom, United States |
| Sub-Saharan Africa (SSA) | Angola, Benin, Botswana, Burkina Faso, Burundi, Cote d’Ivoire, Cameroon, Cape Verde, Central African Republic, Chad, Comoros, Democratic Republic of Congo, Equatorial Guinea, Eritrea, Ethiopia, Gabon, Gambia, Ghana, Guinea, Guinea-Bissau, Kenya, Lesotho, Liberia, Madagascar, Malawi, Mali, Mauritania, Mauritius, Mozambique, Namibia, Niger, Nigeria, Republic of Congo, Rwanda, Senegal, Seychelles, Sierra Leone, Somalia, South Africa, Sudan, Swaziland, Togo, Uganda, Tanzania, Zambia, Zimbabwe |
| Latin America and Caribbean (LAC) | Argentina, Belize, Bolivia, Brazil, Chile, Columbia, Costa Rica, Cuba, Dominican Republic, Ecuador, El Salvador, Guatemala, Guyana, Haiti, Honduras, Jamaica, Mexico, Nicaragua, Panama, Paraguay, Peru, Suriname, Trinidad and Tobago, Uruguay, Venezuela |
| Middle East (MIDEAST) | Algeria, Bahrain, Djibouti, Egypt, Iran, Iraq, Israel, Jordan, Kuwait, Lebanon, Libya, Morocco, Oman, Palestinian Territories, Qatar, Saudi Arabia, Syria, Tunisia, United Arab Emirates, Yemen |
| Eastern Europe and former Soviet Union (EURCA) | Albania, Armenia, Azerbaijan, Belarus, Bosnia, Bulgaria, Croatia, Czech Republic, Estonia, Georgia, Hungary, Kazakhstan, Kyrgyzstan, Latvia, Lithuania, Poland, Moldova, Romania, Russia, Serbia, Slovakia, Slovenia, Tajikistan, Macedonia, Turkey, Turkmenistan, Ukraine, Uzbekistan |
| South Asia (SA) | Afghanistan, Bangladesh, Bhutan, India, Maldives, Nepal, Pakistan, Sri Lanka |
| East Asia and Pacific (EAP) | Cambodia, China, North Korea, Indonesia, Lao PDR, Malaysia, Mongolia, Myanmar, Papua New Guinea, Philippines, South Korea, Thailand, Vietnam |
