# Supplementary material for: The Long-Term Dynamics of Mortality Benefits from Improved Water and Sanitation in Less Developed Countries
Source: PLoS One. 2013 Oct 8;8(10):e74804. doi: 10.1371/journal.pone.0074804 (PMC3792953; doi:10.1371/journal.pone.0074804)
Supplement: Table S3 — Estimation of population coverage with piped water. (DOCX) [file pone.0074804.s007.docx]

Table S3. Estimation of population coverage with piped water.

|  | **Random Effects ^a^** | | | **Fixed Effects** | | | | | |  |  |
| --- | --- | --- | --- | --- | --- | --- | --- | --- | --- | --- | --- |
|  | **Dev. countries only, w/WASH aid** | **Dev. countries only, linear and squared income** | **All countries, linear and squared income** | **Simple model**  **w/WASH aid** | | **Dev. countries only, linear and squared income** | | **All countries, linear and squared income** | | |  |
| 5-yr lagged ln GDP per capita | 11.0*** (3.6) |  |  | | -0.85 (5.2) | |  | |  | | |
| 5-yr lagged linear GDP per capita |  | 0.0054*** (0.00097) | 0.0038*** (0.00053) | |  | | 0.0041*** (0.0010) | | 0.0016*** (0.00045) | | |
| 5-yr lagged squared GDP per capita |  | -2.2e-7*** (4.4e-8) | -1.1e-7*** (1.5e-8) | |  | | -1.8e-7*** (4.8e-8) | | -6.2e-8*** (1.3e-8) | | |
| % of GDP to lowest 80% of population | 0.12 (0.16) | 0.13 (0.10) | 0.043 (0.090) | | 0.09 (0.24) | | 0.13 (0.10) | | 0.13 (0.096) | | |
| % Urban population | 0.47*** (0.12) | 0.45*** (0.09) | 0.71*** (0.073) | | 0.77* (0.46) | | 0.33** (0.17) | | 0.42*** (0.15) | | |
| Countries in LAC region | 30.0*** (5.9) | 29.6*** (5.0) | 15.9*** (3.9) | |  | |  | |  | | |
| Countries in MIDEAST region | 28.0*** (8.4) | 33.9*** (5.5) | 20.8*** (4.8) | |  | |  | |  | | |
| Countries in SOUTH ASIA region | 5.3 (3.5) | 6.3** (3.0) | 0.46 (3.4) | |  | |  | |  | | |
| Countries in EAST ASIA / PACIFIC region | 1.3 (5.1) | 10.3** (5.2) | 1.0 (4.9) | |  | |  | |  | | |
| Countries in EASTERN EUROPE region | 41.5*** (7.3) | 46.1*** (4.0) | 23.7*** (3.1) | |  | |  | |  | | |
| 1990 |  | -4.8*** (1.5) | -0.67 (3.1) | |  | | -6.9*** (1.9) | | -6.5*** (1.7) | | |
| 1995 | -4.5*** (1.5) | -2.8** (1.11) | -0.59 (1.2) | | -5.6*** (1.8) | | -4.4*** (1.4) | | -4.6*** (1.3) | | |
| 2000 | -1.9** (0.89) | -1.2 (0.81) | 0.84 (0.65) | | -2.5*** (0.89) | | -2.4** (0.99) | | -2.6*** (0.94) | | |
| 2005 |  | -0.1 (0.39) | 0.96*** (0.33) | |  | | -0.67 (0.49) | | -0.76 (0.46) | | |
| Democracy-Autocracy Score | -0.34* (0.18) | -0.14 (0.10) | 0.086 (0.092) | | -0.67*** (0.23) | | -0.22** (0.10) | | -0.22** (0.10) | | |
| Years since last regime change | -0.08 (0.06) | -0.0045 (0.045) | 0.065 (0.043) | | -0.17** (0.07) | | -0.013 (0.047) | | -0.014 (0.048) | | |
| Coup | 0.30 (2.3) | -0.84 (1.2) | -0.91 (1.1) | | -0.65 (1.9) | | -0.60 (1.2) | | -0.67 (1.1) | | |
| Aid $– large systems | -0.0031 (0.0058) |  |  | | -0.004 (0.0072) | |  | |  | | |
| Aid $ - Basic systems | -0.015 (0.010) |  |  | | -0.015** (0.0072) | |  | |  | | |
| Aid $ - Policy making | -0.012 (0.027) |  |  | | -0.0043 (0.021) | |  | |  | | |
| Constant | -78.6*** (20.7) | -10.2 (6.4) | -8.4 (6.3) | | 10.4 (28.0) | | 16.3 (11.1) | | 26.2 (11.3) | | |
| Number of observations | 124 | 470 | 634 | 470 | | 470 | | 634 | | | |
| Adjusted R^2^ (overall)  (within)  (between) | 0.889  0.536  0.913 | 0.897  0.553  0.908 | 0.873  0.433  0.882 | 0.774  0.559  0.800 | | 0.763  0.562  0.796 | | 0.705  0.507  0.735 | | | |

*Notes*: *Significant at 90%, **Significant at 95%, ***Significant at 99%. Robust standard errors presented in parentheses, clustered at the country level.

^a^ A random-effects tobit model that allows censoring at 0 and 100% coverage does not yield qualitatively different results.
