# Supplementary material for: The Long-Term Dynamics of Mortality Benefits from Improved Water and Sanitation in Less Developed Countries
Source: PLoS One. 2013 Oct 8;8(10):e74804. doi: 10.1371/journal.pone.0074804 (PMC3792953; doi:10.1371/journal.pone.0074804)
Supplement: Table S4 — Estimation of population coverage with all improved water. (DOCX) [file pone.0074804.s008.docx]

Table S4. Estimation of population coverage with all improved water.

|  | **Random Effects ^a^** | | **Fixed Effects** | |
| --- | --- | --- | --- | --- |
|  | **Simple model** | **Full model^b^** | **Simple model** | **Full model** |
| 5-yr lagged ln GDP per capita | 6.2*** (1.7) | 5.5*** (1.1) | 3.5 (2.4) | 1.4 (1.8) |
| % of GDP to lowest 80% of population | -0.23* (0.12) | -0.22** (0.10) | -0.29** (0.13) | -0.25** (0.12) |
| % Urban population | 0.21*** (0.07) | 0.31*** (0.06) | 0.061 (0.18) | 0.25* (0.15) |
| Countries in LAC region | 10.1*** (3.4) | 5.9*** (2.2) |  |  |
| Countries in MIDEAST region | 11.3** (4.4) | 8.4*** (3.2) |  |  |
| Countries in SOUTH ASIA region | 18.6*** (3.7) | 18.2*** (3.2) |  |  |
| Countries in EAST ASIA / PACIFIC region | 5.4 (4.7) | 3.8 (4.0) |  |  |
| Countries in EASTERN EUROPE region | 16.7*** (3.9) | 10.9*** (1.8) |  |  |
| 1990 | -7.8*** (1.2) | -4.5*** (0.96) | -10.0*** (1.7) | -6.7*** (1.3) |
| 1995 | -5.6*** (0.87) | -3.6** (0.73) | -7.2*** (1.3) | -5.1*** (0.98) |
| 2000 | -3.2*** (0.58) | -1.7*** (0.49) | -4.4*** (0.86) | -3.2*** (0.69) |
| 2005 | -1.3*** (0.30) | -0.69*** (0.26) | -2.0*** (0.45) | -1.5*** (0.37) |
| Democracy-Autocracy Score | 0.17* (0.09) | 0.30*** (0.08) | 0.11 (0.10) | 0.18* (0.10) |
| Years since last regime change | -0.012 (0.03) | -0.0038 (0.02) | 0.014 (0.03) | -0.028 (0.03) |
| Coup | -1.1 (0.91) | -1.5* (0.87) | -0.81 (0.86) | -1.0 (0.81) |
| Constant | 27.0 (12.2) | 27.9*** (9.9) | 65.0*** (19.2) | 72.5*** (16.3) |
| Number of observations | 470 | 634 | 470 | 634 |
| Adjusted R^2^ (overall)  (within)  (between) | 0.696  0.538  0.717 | 0.744  0.449  0.770 | 0.508  0.548  0.614 | 0.600  0.468  0.636 |

*Notes*: *Significant at 90%, **Significant at 95%, ***Significant at 99%. Robust standard errors presented in parentheses, clustered at the country level.

^a^ A random-effects tobit model that allows censoring at 0 and 100% coverage does not yield qualitatively different results.

^b^ Includes all countries (including developed and former Soviet republics dropped from the simple model; as in the other coverage regressions the omitted region is SSA; the omitted year is 2010.
