# Supplementary material for: The Long-Term Dynamics of Mortality Benefits from Improved Water and Sanitation in Less Developed Countries
Source: PLoS One. 2013 Oct 8;8(10):e74804. doi: 10.1371/journal.pone.0074804 (PMC3792953; doi:10.1371/journal.pone.0074804)
Supplement: Table S5 — Estimation of population coverage with all improved sanitation. (DOCX) [file pone.0074804.s009.docx]

Table S5. Estimation of population coverage with all improved sanitation.

|  | **Random Effects ^a^** | | **Fixed Effects** | |
| --- | --- | --- | --- | --- |
|  | **Simple model** | **Full model^b^** | **Simple model** | **Full model** |
| 5-yr lagged ln GDP per capita | 10.4*** (2.2) | 9.2*** (1.6) | 9.3*** (2.5) | 5.6*** (1.9) |
| % of GDP to lowest 80% of population | -0.044 (0.097) | -0.11 (0.096) | -0.31 (0.10) | -0.047 (0.098) |
| % Urban population | 0.37*** (0.12) | 0.54*** (0.10) | 0.44** (0.19) | 0.54*** (0.18) |
| Countries in LAC region | 18.9*** (6.4) | 7.9* (4.7) |  |  |
| Countries in MIDEAST region | 28.0*** (6.7) | 17.4*** (4.5) |  |  |
| Countries in SOUTH ASIA region | 14.7 (9.3) | 9.0 (9.6) |  |  |
| Countries in EAST ASIA / PACIFIC region | 17.8*** (6.7) | 9.9* (5.4) |  |  |
| Countries in EASTERN EUROPE region | 38.1*** (5.2) | 26.9*** (3.9) |  |  |
| 1990 | -5.3*** (1.3) | -1.5 (1.0) | -5.0*** (1.7) | -3.2** (1.5) |
| 1995 | -3.6*** (0.95) | -1.3* (0.78) | -3.5*** (1.3) | -2.6** (1.1) |
| 2000 | -1.7** (0.70) | 0.24 (0.53) | -1.6* (0.91) | -0.98 (0.77) |
| 2005 | -0.29 (0.37) | 0.48* (0.26) | -0.28 (0.49) | -0.16 (0.41) |
| Democracy-Autocracy Score | -0.076 (0.089) | 0.063 (0.085) | -0.095 (0.092) | -0.059 (0.096) |
| Years since last regime change | -0.0056 (0.033) | -0.0062 (0.030) | -0.012 (0.034) | -0.039 (0.036) |
| Coup | 0.33 (0.90) | -0.61 (0.95) | 0.48 (0.90) | -0.36 (0.96) |
| Constant | -51.3*** (14.5) | -40.7*** (11.6) | -33.3 (20.5) | -3.9 (17.8) |
| Number of observations | 470 | 634 | 470 | 634 |
| Adjusted R^2^ (overall)  (within)  (between) | 0.749  0.569  0.760 | 0.751  0.449  0.757 | 0.628  0.571  0.638 | 0.625  0.467  0.623 |

*Notes*: *Significant at 90%, **Significant at 95%, ***Significant at 99%. Robust standard errors presented in parentheses, clustered at the country level.

^a^ A random-effects tobit model that allows censoring at 0 and 100% coverage does not yield qualitatively different results.

^b^ Includes all countries (including developed and former Soviet republics dropped from the simple model; as in the other coverage regressions the omitted region is SSA; the omitted year is 2010.
