# Supplementary material for: The Long-Term Dynamics of Mortality Benefits from Improved Water and Sanitation in Less Developed Countries
Source: PLoS One. 2013 Oct 8;8(10):e74804. doi: 10.1371/journal.pone.0074804 (PMC3792953; doi:10.1371/journal.pone.0074804)
Supplement: Table S6 — Estimation of WASH-related mortality (deaths per thousand people per year); alternative random effects model specifications.a (DOCX) [file pone.0074804.s010.docx]

**Table S6.** Estimation of WASH-related mortality (deaths per thousand people per year); alternative random effects model specifications.^a^

|  | | **Developing**  **countries only,**  **linear model** | | | | **Developing**  **countries only,**  **log coverage terms** | | | | **Developing**  **countries only,**  **higher order terms** | | | | **Developing**  **countries only,**  **linear + log coverage terms** | | | |
| --- | --- | --- | --- | --- | --- | --- | --- | --- | --- | --- | --- | --- | --- | --- | --- | --- | --- |
|  | | **Coef.** | | **Std. Err.^b^** | | **Coef.** | | **Std. Err.^b^.** | | **Coef.** | | **Std. Err.^b^** | | **Coef.** | | **Std. Err.^b^** | |
| % Piped water coverage | | -0.024*** | | -0.0052 | |  | |  | | -0.040*** | | 0.011 | |  | |  | |
| % Improved non-piped water coverage | | -0.027*** | | -0.0056 | |  | |  | | -0.0097 | | 0.011 | | -0.019*** | | 0.0041 | |
| % Improved sanitation coverage | | -0.00013 | | 0.0033 | |  | |  | | -0.0059 | | 0.012 | | 0.0012 | | 0.0080 | |
| Log % Piped water coverage | |  | |  | | -0.38*** | | 0.087 | |  | |  | | -0.53*** | | 0.11 | |
| Log % Improved non-piped water coverage | |  | |  | | -0.24*** | | 0.053 | |  | |  | |  | |  | |
| Log % Improved sanitation coverage | |  | |  | | -0.18 | | 0.16 | |  | |  | | -0.15 | | 0.40 | |
| Squared % Piped water coverage | |  | |  | |  | |  | | 0.00020** | | 0.00013 | |  | |  | |
| Squared % Improved non-piped water coverage | |  | |  | |  | |  | | -0.00016 | | 0.00013 | |  | |  | |
| Squared % Improved sanitation coverage | |  | |  | |  | |  | | 0.00005 | | 0.00009 | |  | |  | |
| Lagged ln per capita GDP | | -0.20*** | | -0.079 | | -0.18** | | 0.090 | | -0.23*** | | 0.073 | | -0.22*** | | 0.080 | |
| % Urban population | | 0.0033 | | 0.0033 | | 0.00047 | | 0.0031 | | 0.0030 | | 0.0033 | | 0.0013 | | 0.0029 | |
| Literacy | |  | |  | |  | |  | |  | |  | |  | |  | |
| % of GDP to lowest 80% of population | |  | |  | |  | |  | |  | |  | |  | |  | |
| Countries in LAC region | | -0.68*** | | 0.13 | | -0.59*** | | 0.17 | | -0.53*** | | 0.15 | | -0.62*** | | 0.16 | |
| Countries in MIDEAST region | | -0.62*** | | 0.17 | | -0.56*** | | 0.22 | | -0.56*** | | 0.18 | | -0.61*** | | 0.19 | |
| Countries in SOUTH ASIA region | | -0.21 | | 0.16 | | -0.48*** | | 0.18 | | -0.13 | | 0.17 | | -0.18 | | 0.17 | |
| Countries in EAST ASIA / PACIFIC region | | -0.73*** | | 0.14 | | -0.70*** | | 0.15 | | -0.67*** | | 0.14 | | -0.62*** | | 0.14 | |
| Countries in EASTERN EUROPE region | | -0.59*** | | 0.15 | | -0.53* | | 0.18 | | -0.54*** | | 0.14 | | -0.57*** | | 0.16 | |
| Democracy-Autocracy Score | | 0.0016 | | 0.0058 | | -0.0044 | | 0.0056 | | -0.00082 | | 0.0055 | | 0.0034 | | 0.0051 | |
| Years since last regime change | | 0.0017 | | 0.0023 | | 0.0021 | | 0.0023 | | 0.0017 | | 0.0023 | | 0.0014 | | 0.0023 | |
| 2004 | | 0.042 | | 0.034 | | 0.029 | | 0.035 | | 0.038 | | 0.034 | | 0.037 | | 0.034 | |
| 2008 | | -0.20*** | | -0.079 | | -0.14*** | | 0.038 | | -0.095*** | | 0.032 | | -0.097*** | | 0.032 | |
| Constant | | 4.4*** | | 0.67 | | 5.2*** | | 0.65 | | 4.5*** | | 0.62 | | 5.6*** | | 1.1 | |
| Number of observations | | 382 | | | | 367 | | | | 382 | | | | 382 | | | |
| R^2^ (within) | | 0.172 | | | | 0.186 | | | | 0.189 | | | | 0.203 | | | |
| R^2^ (between) | | 0.722 | | | | 0.695 | | | | 0.736 | | | | 0.728 | | | |
| R^2^ (overall) | | 0.679 | | | | 0.654 | | | | 0.693 | | | | 0.686 | | | |

*Notes*: *Significant at 90%, **Significant at 95%, ***Significant at 99%. Robust standard errors presented in parentheses, clustered at the country level. The omitted region in these regressions is Sub-Saharan Africa (SSA); the omitted year is 2002.
