# Supplementary material for: The Long-Term Dynamics of Mortality Benefits from Improved Water and Sanitation in Less Developed Countries
Source: PLoS One. 2013 Oct 8;8(10):e74804. doi: 10.1371/journal.pone.0074804 (PMC3792953; doi:10.1371/journal.pone.0074804)
Supplement: Table S7 — Estimation of DALYs due to WASH-related illnesses (DALYs per thousand people).a (DOCX) [file pone.0074804.s011.docx]

**Table S7.** Estimation of DALYs due to WASH-related illnesses (DALYs per thousand people).^a^

|  | **All Countries,**  **base model**  **(random effects)^c.d^** | | **All Countries,**  **base model**  **(fixed effects)^d^** | | **Developing**  **countries only,**  **base model**  **(random effects)** | | **All Countries,**  **full model**  **(random effects)** | |
| --- | --- | --- | --- | --- | --- | --- | --- | --- |
|  | **Coef.** | **Std. Err.^b^.** | **Coef.** | **Std. Err.^b^** | **Coef.** | **Std. Err.^b^** | **Coef.** | **Std. Err.^b^** |
| % Piped water coverage | -0.60*** | 0.18 | 0.032 | 0.75 | -0.62*** | 0.20 | -0.58* | 0.31 |
| % Improved non-piped water coverage | -0.66*** | 0.21 | 0.26 | 0.75 | -0.68*** | 0.22 | -0.56* | 0.33 |
| % Improved sanitation coverage | 0.12 | 0.13 | -0.49 | 0.73 | 0.15 | 0.14 | 0.18 | 0.17 |
| Lagged ln per capita GDP | -10.1*** | 2.6 | -13.2 | 14.3 | -11.5*** | 2.9 | -8.5** | 3.8 |
| % Urban population | 0.15 | 0.12 | -2.5 | 1.8 | 0.16 | 0.12 | 0.16 | 0.15 |
| Literacy |  |  |  |  |  |  | -0.26** | 0.12 |
| % of GDP to lowest 80% of population |  |  |  |  |  |  | 0.079 | 0.32 |
| Developed countries | -7.8* | 4.8 |  |  | -6.1* | 4.6 | -2.6 | 5.7 |
| Countries in LAC region | -18.3*** | 4.4 |  |  | -17.7*** | 4.2 | -15.2** | 7.2 |
| Countries in MIDEAST region | -15.4** | 6.3 |  |  | -14.3** | 6.5 | -17.5** | 7.3 |
| Countries in SOUTH ASIA region | -3.2 | 6.8 |  |  | -2.6 | 7.1 | -11.6* | 7.0 |
| Countries in EAST ASIA / PACIFIC region | -19.3*** | 4.0 |  |  | -18.9*** | 4.1 | -16.9*** | 5.1 |
| Countries in EASTERN EUROPE region | -18.5*** | 4.9 |  |  | -17.0*** | 4.7 | -12.9** | 5.8 |
| Democracy-Autocracy Score | 0.17 | 0.22 | -0.41 | 0.97 | 0.22 | 0.27 | 0.13 | 0.26 |
| Years since last regime change | -0.0009 | 0.026 | -1.1 | 0.79 | -0.0035 | 0.027 | -0.023 | 0.023 |
| 2004 | 2.1** | 0.82 | 5.5** | 2.2 | 1.7* | 0.89 | 2.8*** | 0.82 |
| Constant | 141.5*** | 22.8 | 300.7 | 188.2 | 150.9*** | 25.2 | 135.5*** | 25.5 |
| Number of observations | 300 | | 300 | | 270 | | 249 | |
| R^2^ (within) | 0.027 | | 0.089 | | 0.022 | | 0.033 | |
| R^2^ (between) | 0.706 | | 0.369 | | 0.703 | | 0.721 | |
| R^2^ (overall) | 0.678 | | 0.357 | | 0.675 | | 0.692 | |

*Notes*: *Significant at 90%, **Significant at 95%, ***Significant at 99%

^a^ The omitted region in these regressions is Sub-Saharan Africa (SSA); the omitted year is 2002. ^b^ Robust standard errors.

^c^ A Breusch-Pagan specification test is highly significant (P-value<0.000), indicating that the random effects model is more efficient than a standard OLS specification.

^d^ Hausman tests indicate that we cannot reject the hypothesis that the model estimates obtained from random effects and OLS (P-value = 1.0), and fixed effects and OLS (P-value = 0.75) specifications, are systematically different. The Hausman test however suggests that random effects and fixed effects model estimates are different (P-value = 0.0011), but the same caveats apply as in footnote 6.
